# Comprehensive RNA velocity by modeling the cascade of gene regulation, transcription, and splicing from single-cell RNA sequencing data with TSvelo

**DOI:** 10.7554/eLife.108950

**Published:** 2026-09-15

**Authors:** Jiachen Li, Zhe Wang, Hong-Bin Shen, Ye Yuan

**Affiliations:** 1 State Key Laboratory of Biopharmaceutical Preparation and Delivery, Institute of Process Engineering, Chinese Academy of Sciences Beijing China; 2 Institute of Image Processing and Pattern Recognition, Shanghai Jiao Tong University, and Key Laboratory of System Control and Information Processing, Ministry of Education of China Shanghai China; Shanghai University of Medicine and Health Sciences China; CNRS France

**Keywords:** RNA velocity, cell trajectory, scRNA-seq analysis, Mouse

## Abstract

RNA velocity approaches fit gene dynamics and infer cell fate by modeling the splicing process using single-cell RNA sequencing (scRNA-seq) data. However, due to the short time scale of splicing, high noise, and large complexity of data, existing RNA velocity methods often fail to precisely capture the complex velocity dynamics for individual genes and single cells, which makes their downstream analysis less reliable and less robust. We propose **TSvelo**, a comprehensive RNA **velo**city mathematics framework that can model the cascade of gene regulation, **T**ranscription and **S**plicing using highly interpretable neural ordinary differential equations. TSvelo can precisely capture the transcription–unspliced–spliced 3D dynamics of all genes simultaneously, infer unified latent time shared by genes within a single cell, and be applied to multi-lineage datasets. Experiments on six scRNA-seq datasets, including two multi-lineage datasets, demonstrate TSvelo’s superiority.

## Introduction

Single-cell RNA sequencing (scRNA-seq) enables the detailed exploration of gene expression at the individual cell level. To move beyond static snapshots and capture the dynamic behavior of cells over time, several trajectory inference methods have been developed, such as PAGA (Wolf et al., 2019), Monocle (Qiu et al., 2017), Slingshot (Street et al., 2018), and Palantir (Setty et al., 2019). These methods typically estimate pseudotime using diffusion processes and require prior annotation of initial cells. In contrast, RNA velocity (La Manno et al., 2018) offers a more interpretable approach by modeling the time derivative of gene expression, linking unspliced (immature) and spliced (mature) mRNA levels through ordinary differential equations (ODEs).

Several RNA velocity methods have been proposed to capture splicing dynamics. The first approach, Velocyto (La Manno et al., 2018), uses least squares solutions to estimate parameters under the assumption of steady-state kinetics. ScVelo (Bergen et al., 2020) improves upon this by employing an expectation–maximization (EM) approach for better fitting splicing kinetics. UniTVelo (Gao et al., 2022) proposes a top-down idea that directly models the spliced RNA levels with a time-dependent function. More recently, generative models such as VeloVI (Gayoso et al., 2024), veloVAE (Gu et al., 2022), Pyrovelocity (Qin et al., 2022), and BayVel (Sabbioni et al., 2025) have been introduced, utilizing Bayesian frameworks to estimate RNA velocity. To handle multi-lineage datasets, methods like CellDancer (Li et al., 2024b), DeepVelo (Cui et al., 2024), and LatentVelo (Farrell et al., 2023) extend RNA velocity by modeling local dynamics with neural networks, rather than assuming a globally constant transcriptional rate. Apart from using unspliced/spliced data, Dynamo (Qiu et al., 2022) enhances RNA velocity further by incorporating labeled RNA-seq data. The advent of single-cell multi-omics (Ma et al., 2020; Subramanian et al., 2020) technologies has allowed RNA velocity analysis to extend to protein abundance (e.g., protaccel Gorin et al., 2020) and single-cell ATAC-seq datasets (e.g., MultiVelo Li et al., 2023). Additionally, methods like DeepCycle (Riba et al., 2022) and VeloCycle (Lederer et al., 2024) have been developed to focus on cell cycle processes, with specialized modules for capturing periodic signals. STT (Zhou et al., 2024) and SIRV (Abdelaal et al., 2024) proposed the idea of extending RNA velocity analysis to spatial transcriptomics.

Although RNA velocity theory has significantly advanced the inference of single-cell trajectories, pseudotime, and gene regulation (Liu et al., 2022), several challenges persist for current RNA velocity models. First, RNA velocity models primarily infer cell fate based on phase portrait fitting of unspliced and spliced dynamics for each gene. However, they often fail to capture the correct phase portrait for most genes, due to the sparsity and noise in unspliced and spliced mRNA abundance for individual genes, the short time scale of the splicing process, and the mixing of cells from different types on the phase portrait (Gorin et al., 2022; Soneson et al., 2021; Li et al., 2024a). Second, the majority of existing RNA velocity models treat each gene independently and fail to incorporate the underlying regulatory interactions (Bergen et al., 2021). Although some approaches (e.g., TFvelo Li et al., 2024a, PHOENIX Hossain et al., 2024, scKINETICS Burdziak et al., 2023, and scPN Zhou et al., 2025) have constructed ODE models to capture gene dynamics by integrating regulation information, these methods overlook the splicing signal so that they cannot jointly model the transcription and splicing dynamics into one unified form. Third, classical RNA velocity approaches, such as scVelo, use interpretable parameters in constructing dynamic models for single genes. By contrast, to model flexible transcriptional rates or integrate multiple genes, several recent methods employ latent space embeddings or neural network-based encoders (Li et al., 2024b). This makes the model parameters less interpretable at the detailed gene level, which is crucial for understanding the underlying biological mechanisms. Fourth, multi-lineage tasks remain a significant challenge for current RNA velocity models due to the complexity in large-scale scRNA-seq datasets.

To address the challenges outlined above, we propose TSvelo, a method that integrates gene regulation, transcription, and splicing of all genes into a single ODE model, whose parameters are highly interpretable. Using a high-dimensional Neural ODE solver (Chen et al., 2018; Gao et al., 2025), TSvelo directly learns the global latent time without the need to separately learn gene-specific latent times. By leveraging both unspliced and spliced scRNA-seq data, along with gene regulatory knowledge from transcription factor (TF)–target databases, TSvelo iteratively optimizes the parameters in the ODE model and the unified latent time using the EM algorithm. Experiments on six scRNA-seq datasets, including two multi-lineage datasets, demonstrate that TSvelo outperforms existing methods in modeling gene dynamics, inferring cell fates, and is also effective in analyzing multi-lineage datasets.

## Results

### Estimate RNA velocity with TSVelo

**Figure 1.**
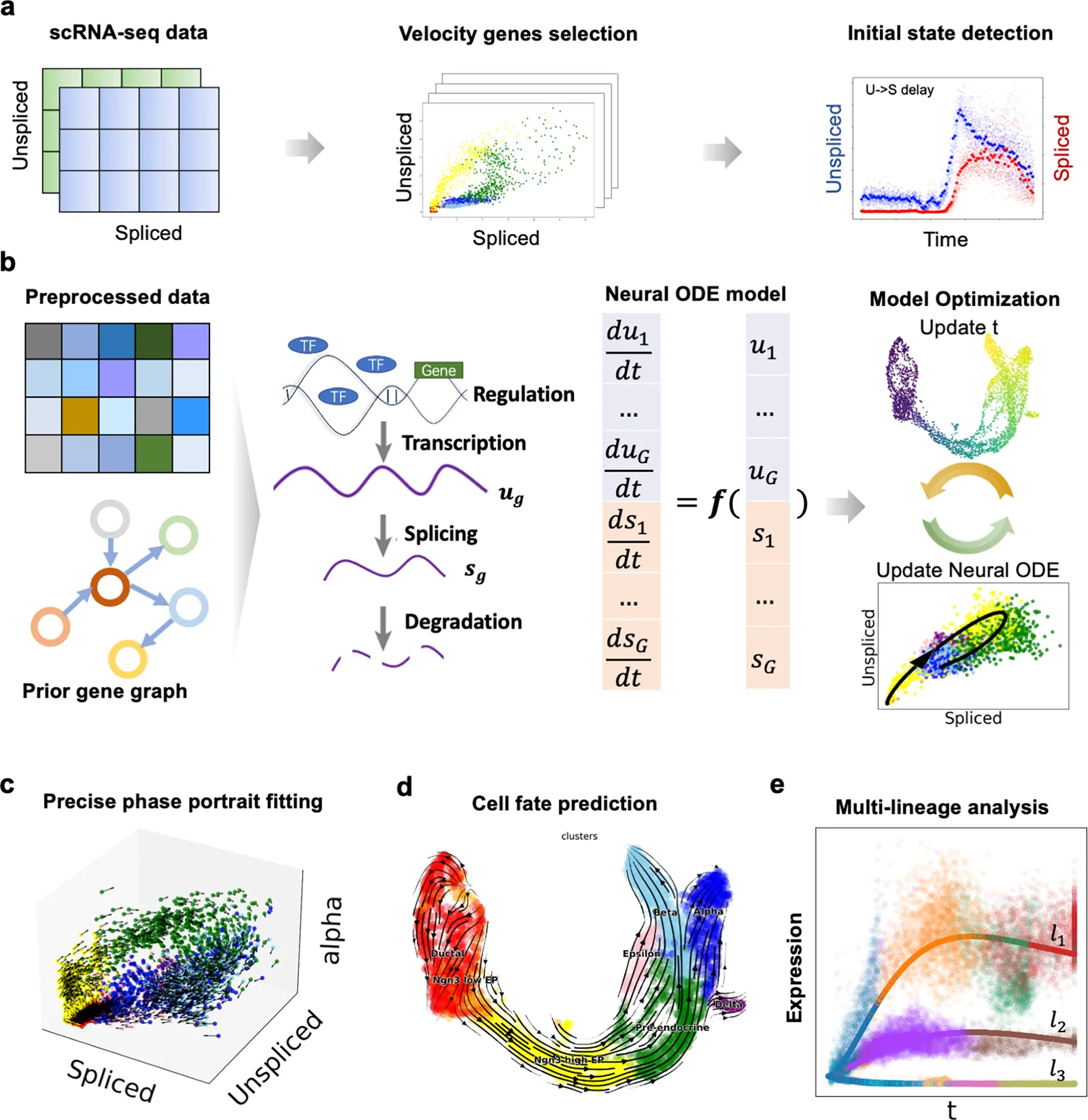
The framework of TSvelo. (**a**) The preprocessing strategy in TSvelo, including velocity genes selection and initial state detection. (**b**) The neural ordinary differential equation (ODE) model and its optimization in TSvelo, where the parameters and latent time are optimized iteratively. The downstream application tasks of TSvelo, including precise transcription–unspliced–spliced 3D phase portrait fitting (**c**), cell fate prediction using predicted RNA velocity (**d**), and gene expression pattern analysis for multi-lineage dataset (**e**).

We present TSvelo, a method for jointly estimating RNA velocity across high-dimensional genes by integrating both transcriptional regulation and splicing information. Existing RNA velocity models predominantly focus on modeling the splicing process and typically operate in a gene-wise manner. In contrast, TSvelo explicitly models the full cascade of regulation, transcription, and splicing, while capturing their coordinated dynamics across all genes simultaneously. A systematic comparison between TSvelo and representative RNA velocity methods is provided in Appendix 1—table 1. In TSvelo, the unspliced and spliced RNA abundances are initially preprocessed for velocity gene selection and pseudotime initialization (Figure 1a). See **Methods** for details about preprocessing. Next, to model the velocity gene g’s expression dynamics, we suppose $u_{g}$ and $s_{g}$ are the abundance of unspliced and spliced RNA, and $\alpha _{g}\left (t\right),\beta _{g}$, and $\gamma _{g}$ are the transcription, splicing, and degradation rates, respectively. The dynamics is modeled as

(1) $$\displaystyle \frac{du_{g}\left (t\right)}{dt}=\alpha _{g}\left (t\right)- \beta _{g}u_{g}\left (t\right)$$

(2) $$\displaystyle \frac{ds_{g}\left (t\right)}{dt}=\beta _{g}u_{g}\left (t\right)- \gamma _{g}s_{g}\left (t\right)$$

Furthermore, we assume that the gene- and cell-specific transcriptional rate $\alpha _{g}\left (t\right)$ is influenced by the expression of TFs.

**Figure 1—figure supplement 1.**
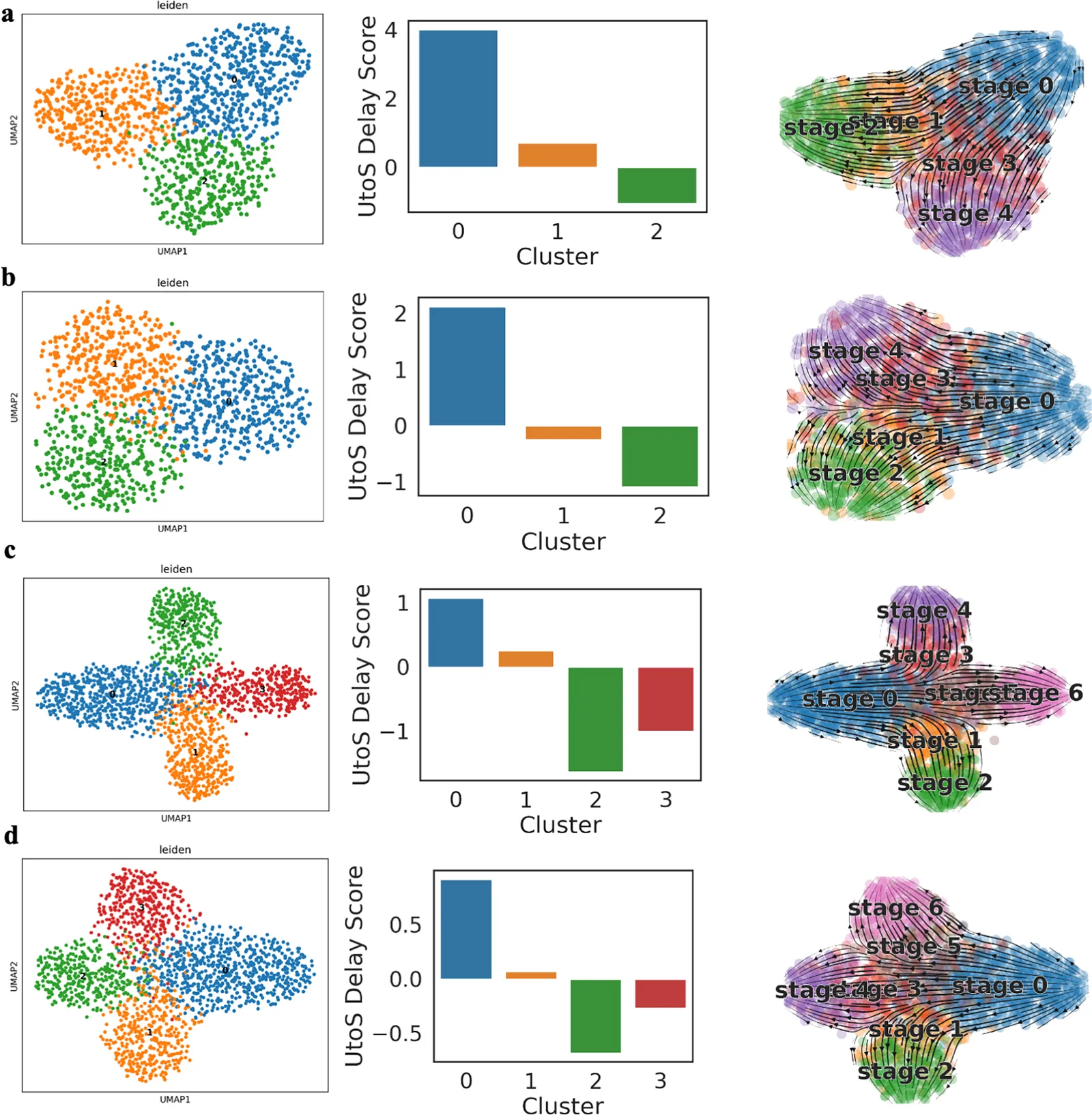
Initial state detection and the 2D velocity stream obtained by TSvelo on multiple simulation datasets.

**Figure 1—figure supplement 2.**
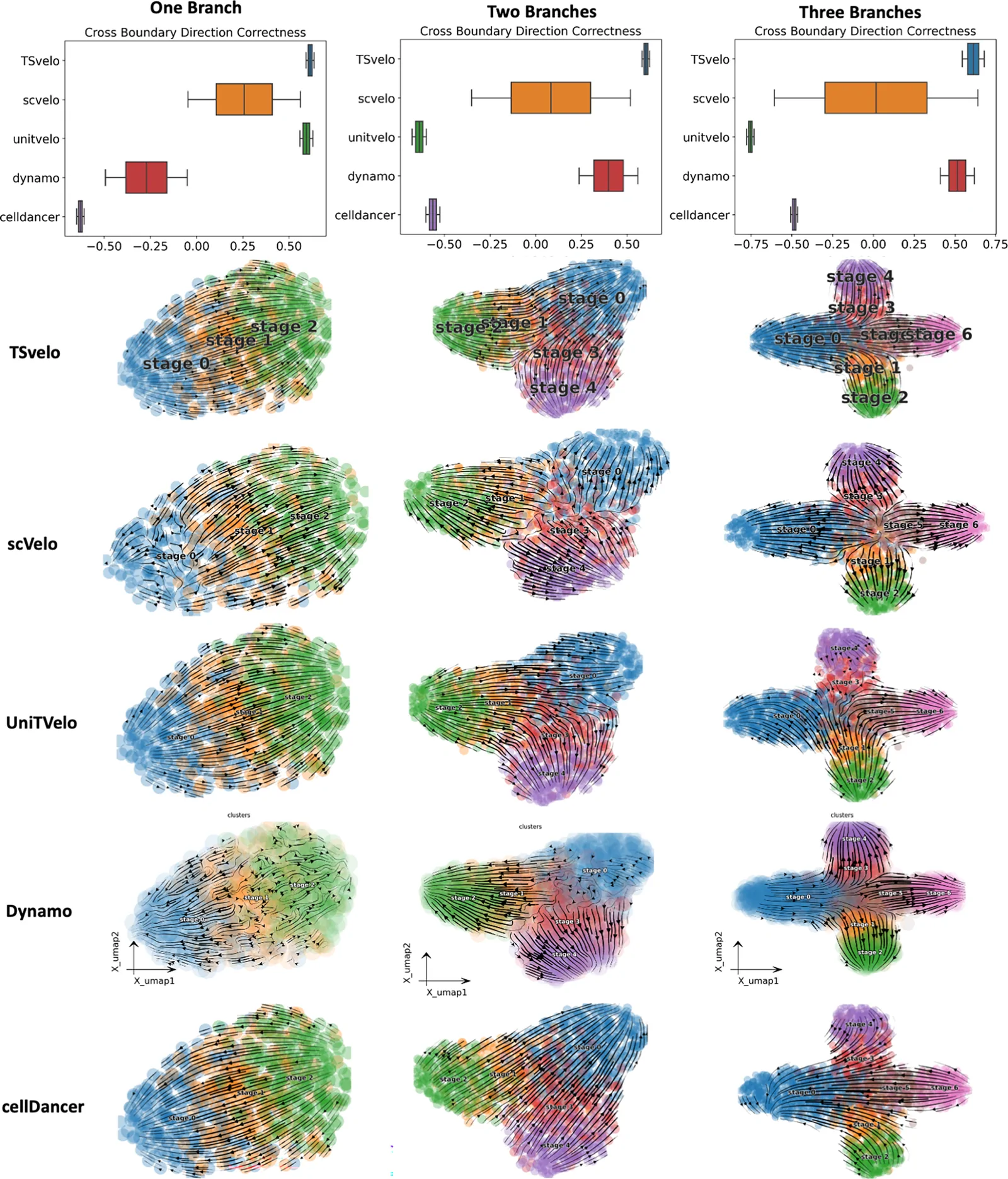
Comparison between TSvelo and baseline approaches using the simulation data. For these boxplots in the top row, the whiskers extend to 1.5× the interquartile range of the distribution from the hinge. The sample sizes are 600, 1200 and 1800 for one branch, tow branches and three branches respectively.

**Figure 1—figure supplement 3.**
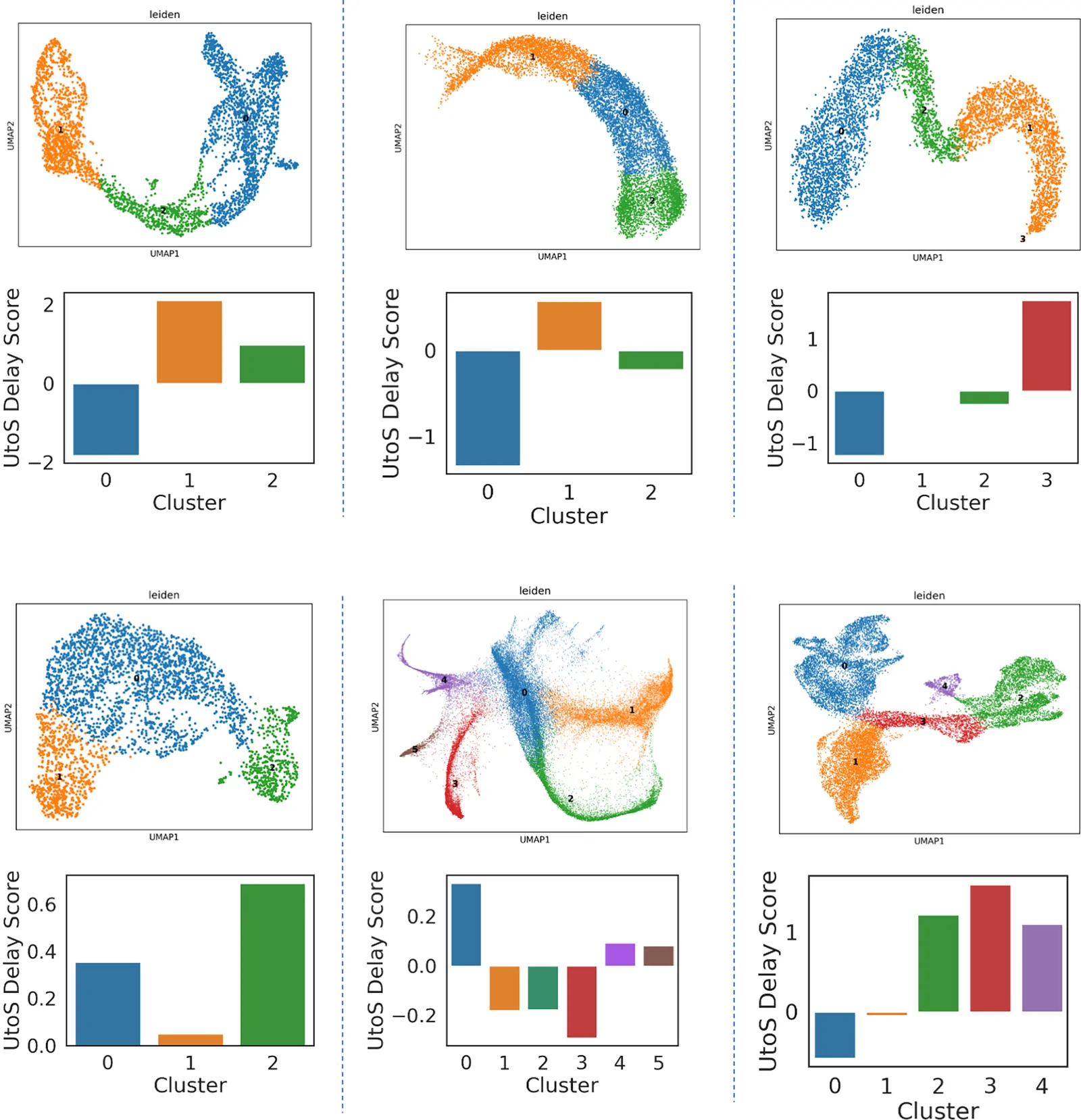
The U-to-S delay scores for each Leiden cluster when treated as the initial state across all datasets.

**Figure 1—figure supplement 4.**
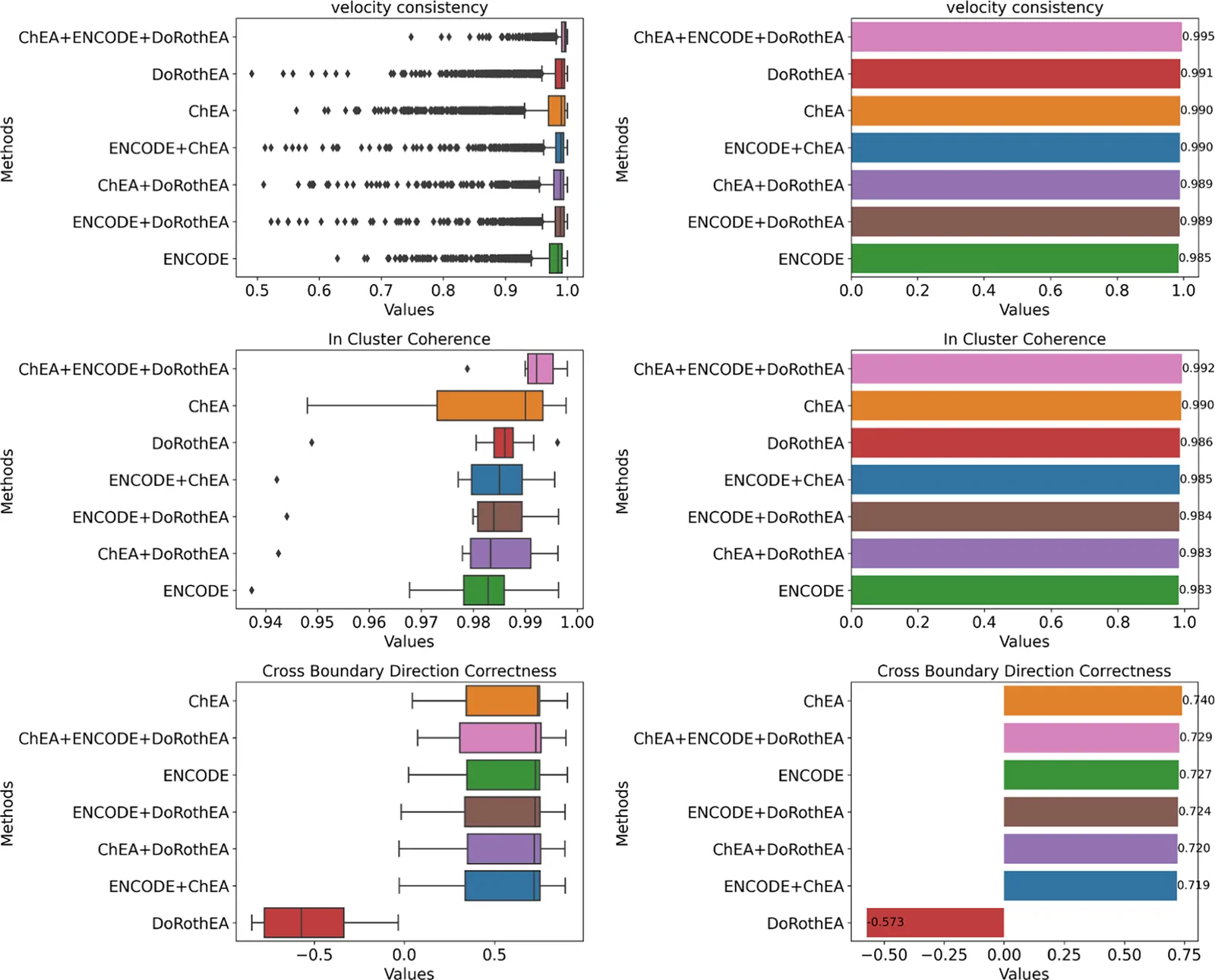
The quantitative comparison of TSvelo modeling results on the pancreas dataset using different transcription factor (TF)–target prior databases. The panels in the left column show the boxplot of velocity consistency, the in-cluster coherence, and the cross-boundary direction correctness, respectively. The panels in the right column show the barplot of their mean values. For these boxplots in the left column, the whiskers extend to 1.5× the interquartile range of the distribution from the hinge. 3696 samples are included in the boxplot for each method.

**Figure 1—figure supplement 5.**
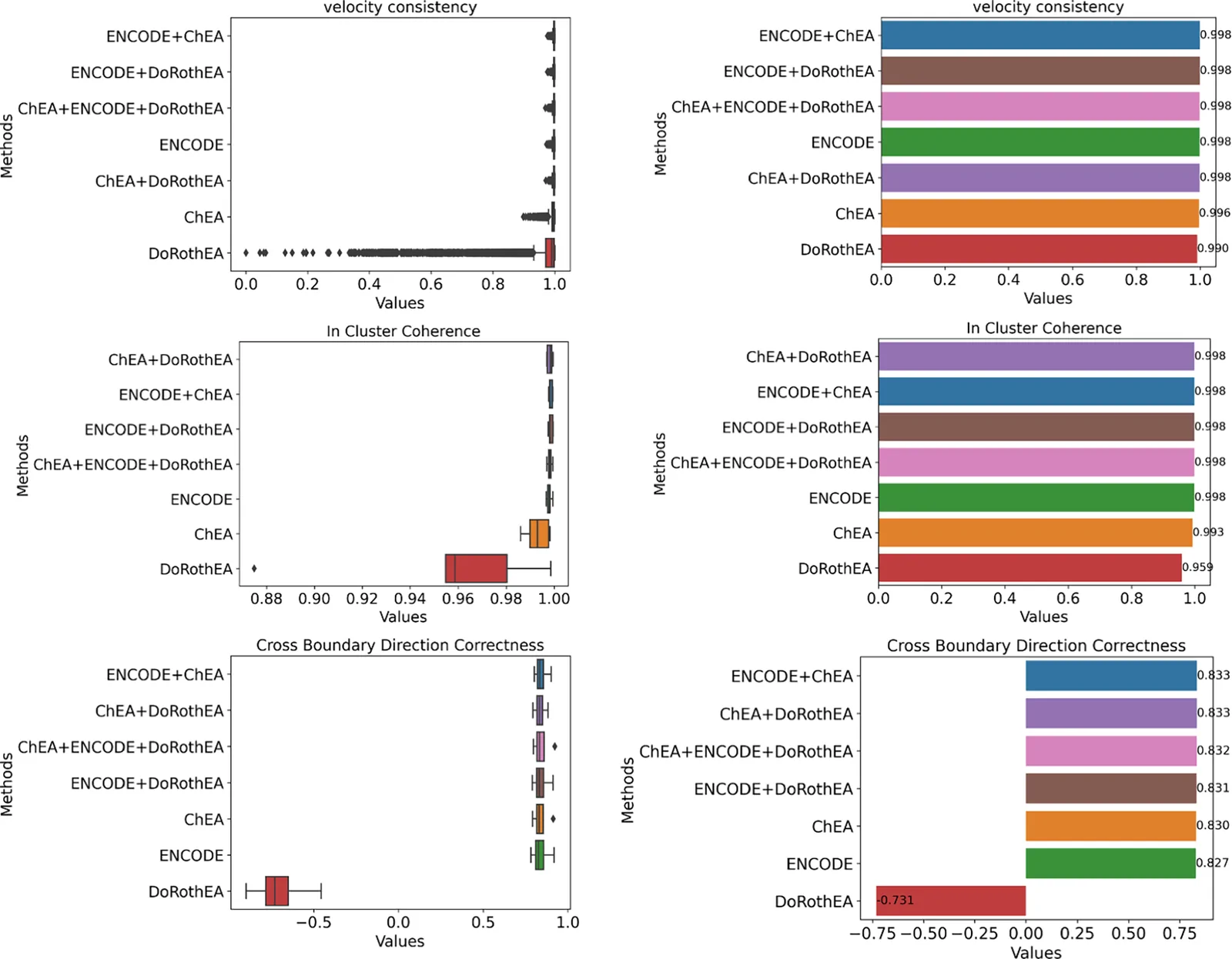
The quantitative comparison of TSvelo modeling results on the gastrulation erythroid dataset using different transcription factor (TF)–target prior databases. The panels in the left column show the boxplot of velocity consistency, the in-cluster coherence, and the cross-boundary direction correctness, respectively. The panels in the right column show the barplot of their mean values. For these boxplots in the left column, the whiskers extend to 1.5× the interquartile range of the distribution from the hinge. 9815 samples are included in the boxplot for each method.

**Figure 1—figure supplement 6.**
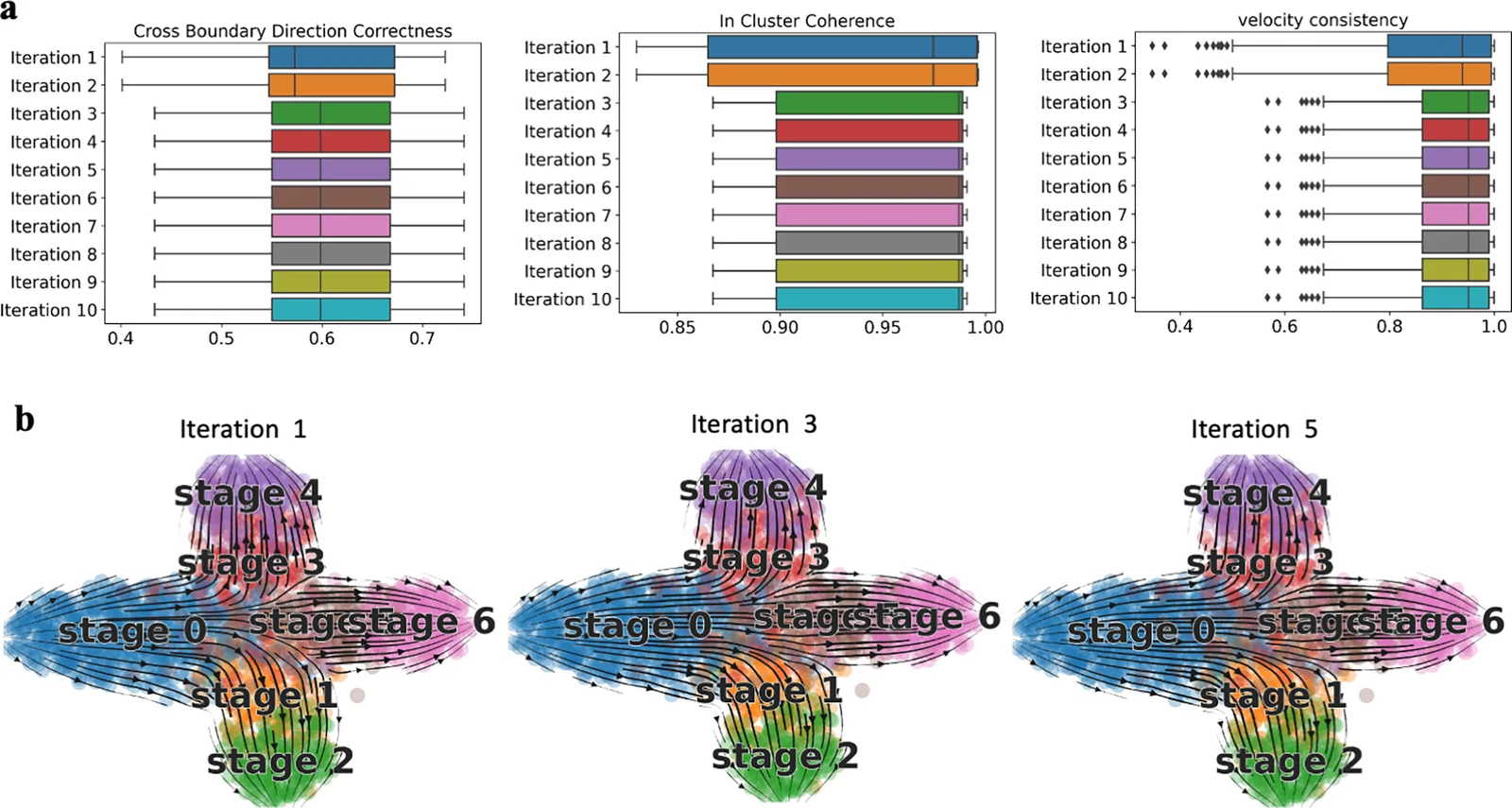
Effect of the number of expectation–maximization (EM) iterations on TSvelo performance in the simulation dataset. (**a**) Quantitative evaluation across different iteration settings. (**b**) 2D visualization of inferred dynamics. The results show that TSvelo converges rapidly, and performance remains stable after three iterations due to the built-in early stopping mechanism in the EM framework. For these boxplots in the top row, the whiskers extend to 1.5× the interquartile range of the distribution from the hinge. The sample size is 1800.

**Figure 1—figure supplement 7.**
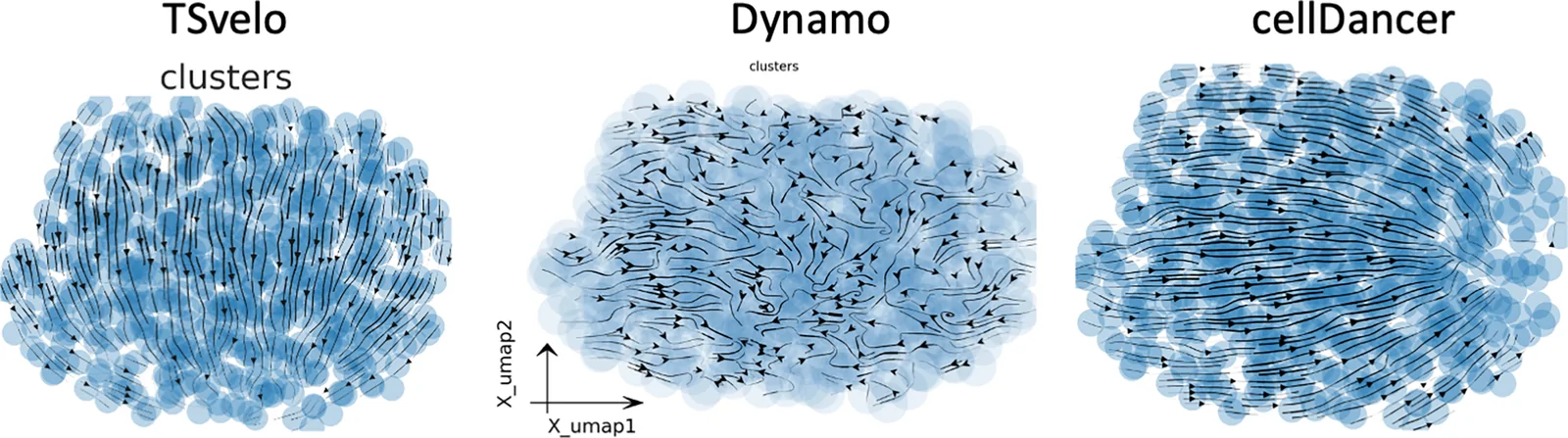
The predicted velocity streams of TSvelo, Dynamo, and cellDancer on the null simulation data.

Considering the wide usage and interpretability of linear models for gene relations in previous studies (Li et al., 2024a; Tran et al., 2022; Song et al., 2023; Liu et al., 2011; Wang et al., 2023), we model $\alpha _{g}\left (t\right)$ as

(3) $$\displaystyle  \alpha _{g}\left (t\right)=ReLU\left (\sum _{i\in TFs\left (g\right)}w_{gi}\ast s_{i}\left (t\right)\right)$$

ReLU denotes the Rectified Linear Unit activation function, defined as $ReLU\left (x\right)=max\left (0,x\right)$. The term $w_{gi}$ represents the regulatory weight of TF i on the target gene g. $TFs\left (g\right)$ refers to the TFs that could regulate the target gene g, which are selected according to the ChEA and ENCODE TF–target databases. In order to include more TFs in the model, we also reserve those TFs that are not selected as velocity genes and directly model the dynamics between transcription and spliced RNA without using the unspliced abundance. Finally, we combine the dynamic models on all selected genes into the ODE matrix form (Figure 1b), which enables directly inferring a unified latent time for each cell using all its genes. See **Methods** for details.

TSvelo employs an EM framework to iteratively optimize both latent time t and the parameters in ODE. The global pseudotime t is assigned to each cell through grid search. Due to the difficulty in calculating analytical solutions of $u_{g}\left (t\right)$ or $s_{g}\left (t\right)$, TSvelo adopted a Neural ODE for estimating those parameters of transcription rate, splicing rate, and degradation rate. Next, TSvelo can model the gene dynamics of both transcription and splicing processes (Figure 1c), predict cell states using pseudotime and velocity stream analysis (Figure 1d), and is also applicable to multi-lineage tasks for analyzing expression patterns across different lineages in large complex scRNA-seq datasets (Figure 1e). We further validated TSvelo on simulated datasets and observed consistent improvements in velocity estimation and trajectory reconstruction across a range of settings (Figure 1—figure supplements 1 and 2).

### TSvelo can model 3D gene dynamics and predict cell fate on pancreas dataset

**Figure 2.**
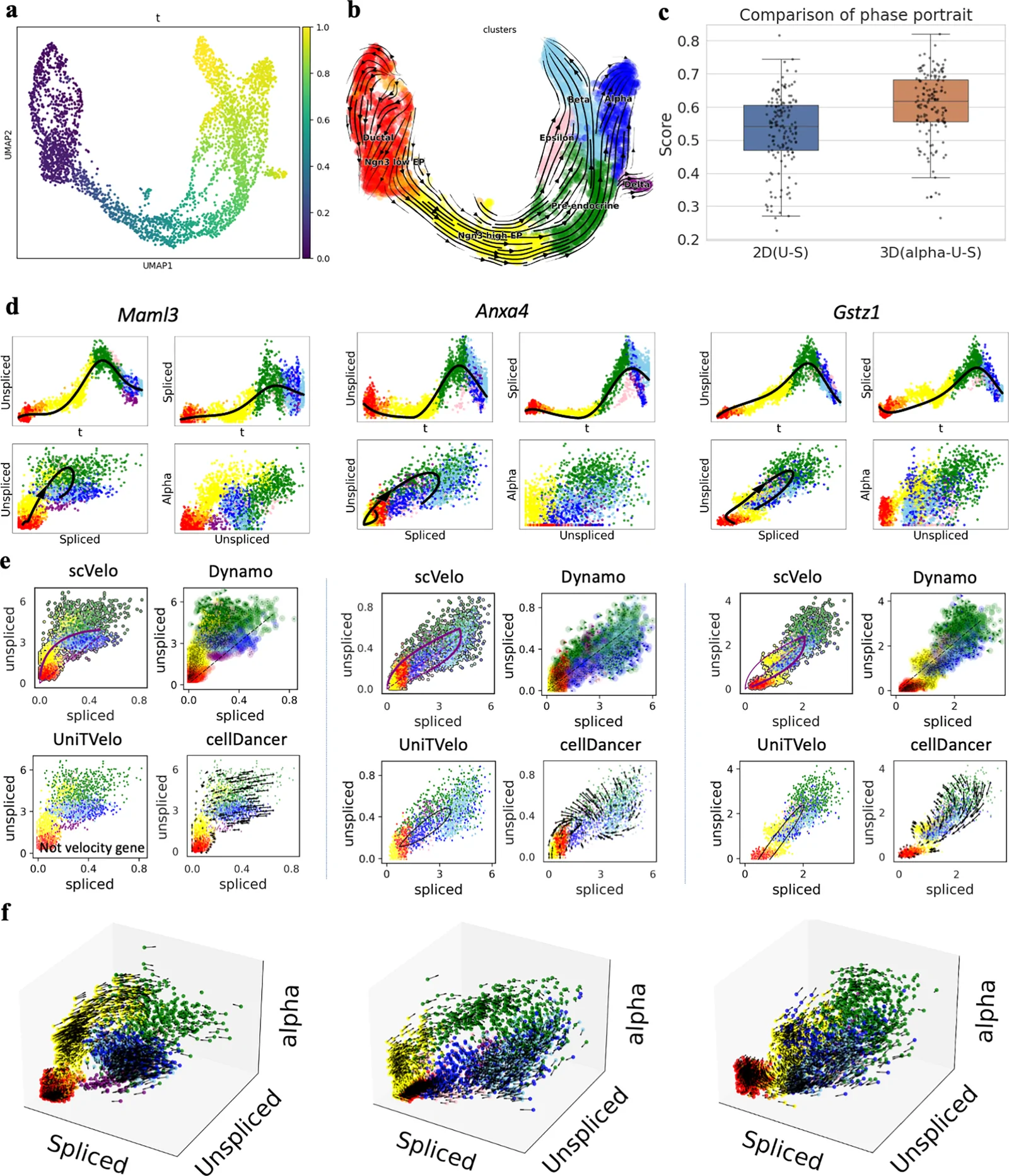
Results on pancreas dataset. (**a**) The pseudotime learned with TSvelo. (**b**) The stream plot for visualizing the RNA velocity inferred by TSvelo. (**c**) A quantitative comparison of cell-state separability between the 3D phase portrait used in TSvelo and the traditional 2D phase portrait. The down, central, and up hinges correspond to the first quartile, median value, and third quartile, respectively. The whiskers extend to 1.5× the interquartile range of the distribution from the hinge. 3696 samples are included in the boxplot for each method. (**d**) The dynamics fitting on *Maml3*, *Anxa4*, and *Gstz1*. For each gene, four plots are displayed in a 2 × 2 layout: the u–t, s–t, u–s, and alpha–u plots. (**e**) The unspliced–spliced phase portrait fitting on *Maml3*, *Anxa4*, and *Gstz1* obtained by four baseline RNA velocity approaches, including scVelo, Dynamo, UniTVelo, and cellDancer. (**f**) The dynamics fitting in transcription–unspliced–spliced 3D phase portrait for *Maml3*, *Anxa4*, and *Gstz1*. Figure 2—source data 1.Source data of the comparison between the 2D and 3D phase portrait fitting.

To validate the TSvelo model, we applied it to the pancreas scRNA-seq dataset (Bastidas-Ponce et al., 2019), which is widely used in RNA velocity studies to model cell differentiation from ductal to endocrine cells (La Manno et al., 2018). Using the predicted RNA velocity, TSvelo can generate velocity stream plot using the functions provided in scVelo as well. Both the pseudotime t (Figure 2a) and the velocity stream plot (Figure 2b) effectively capture the cell differentiation process. We quantitatively compare TSvelo and baseline approaches, including scVelo (Bergen et al., 2020), dynamo (Qiu et al., 2022), UniTVelo (Gao et al., 2022), cellDancer (Li et al., 2024b), and TFvelo (Li et al., 2024a).

**Figure 2—figure supplement 1.**
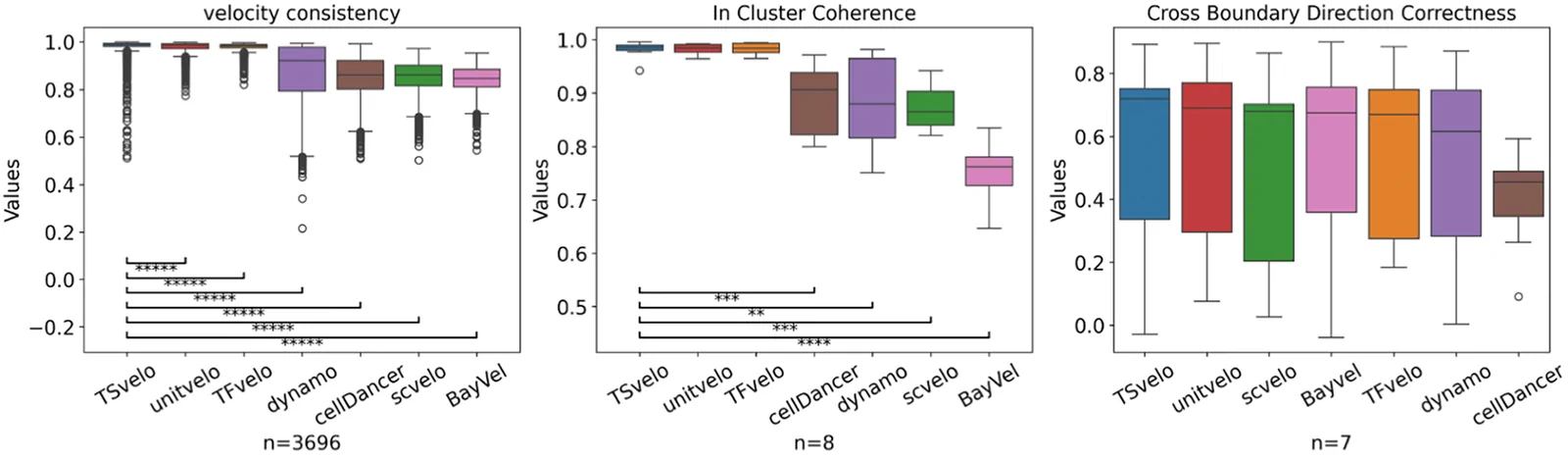
The velocity consistency, in-cluster coherence, and cross-boundary direction correctness on pancreas dataset. The whiskers extend to 1.5× the interquartile range of the distribution from the hinge. 3696 samples are included in the boxplot for each method. In each plot, methods are ranked in descending order of their mean values. Numbers at the bottom indicate the sample size for each metric. Significance is determined using a one-sided Mann–Whitney *U* test. *****, ****, ***, **, and * represent p < 0.00001, 0.00001 ≤ p < 0.0001, 0.0001 ≤ p < 0.001, 0.001 ≤ p < 0.01, and 0.01 ≤ p < 0.05, respectively.

**Figure 2—figure supplement 2.**
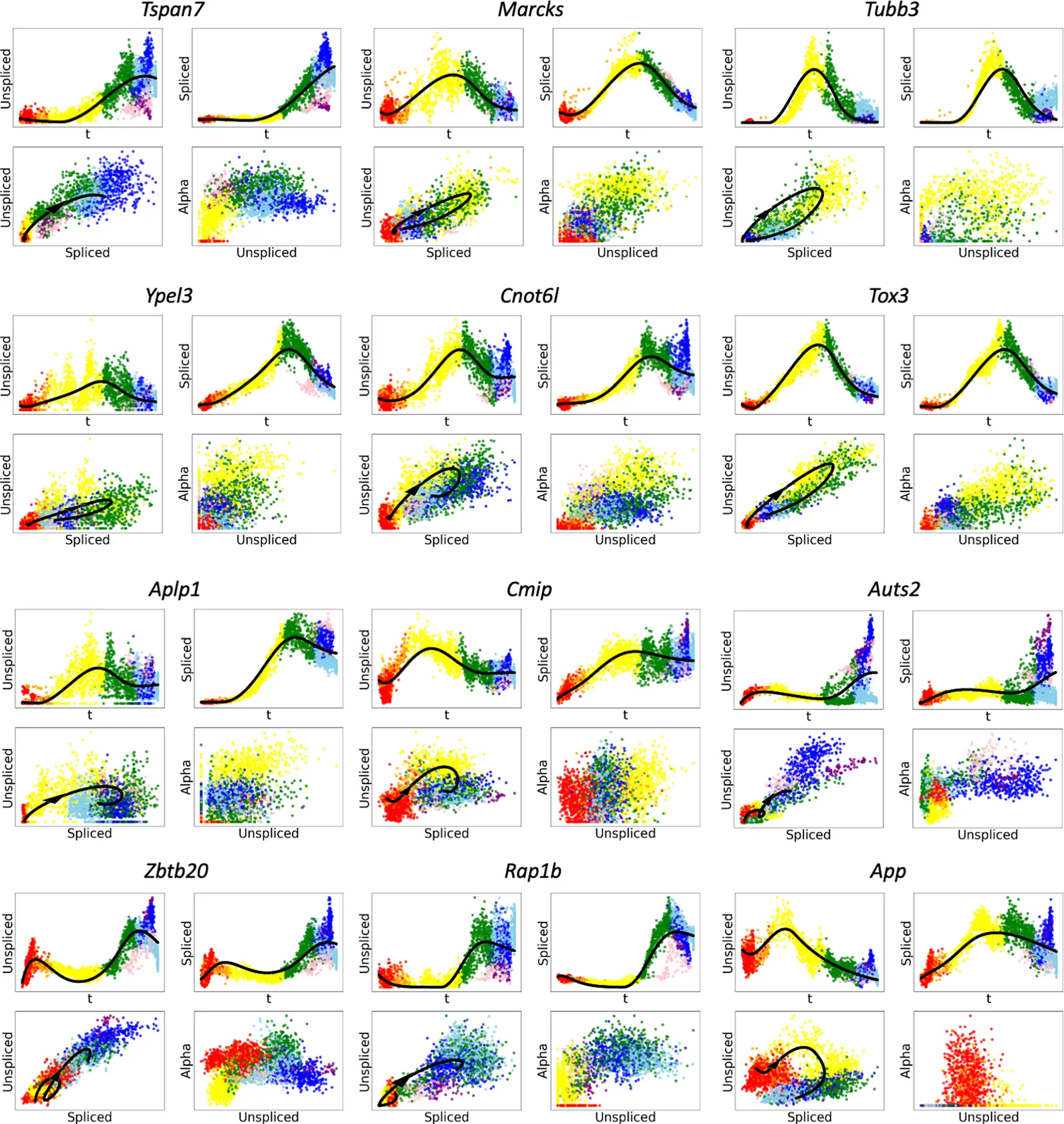
Dynamics fitting of velocity genes on the pancreas dataset. For each gene, four plots are displayed in a 2 × 2 layout: the u–t, s–t, u–s, and alpha–u plots.

**Figure 2—figure supplement 3.**
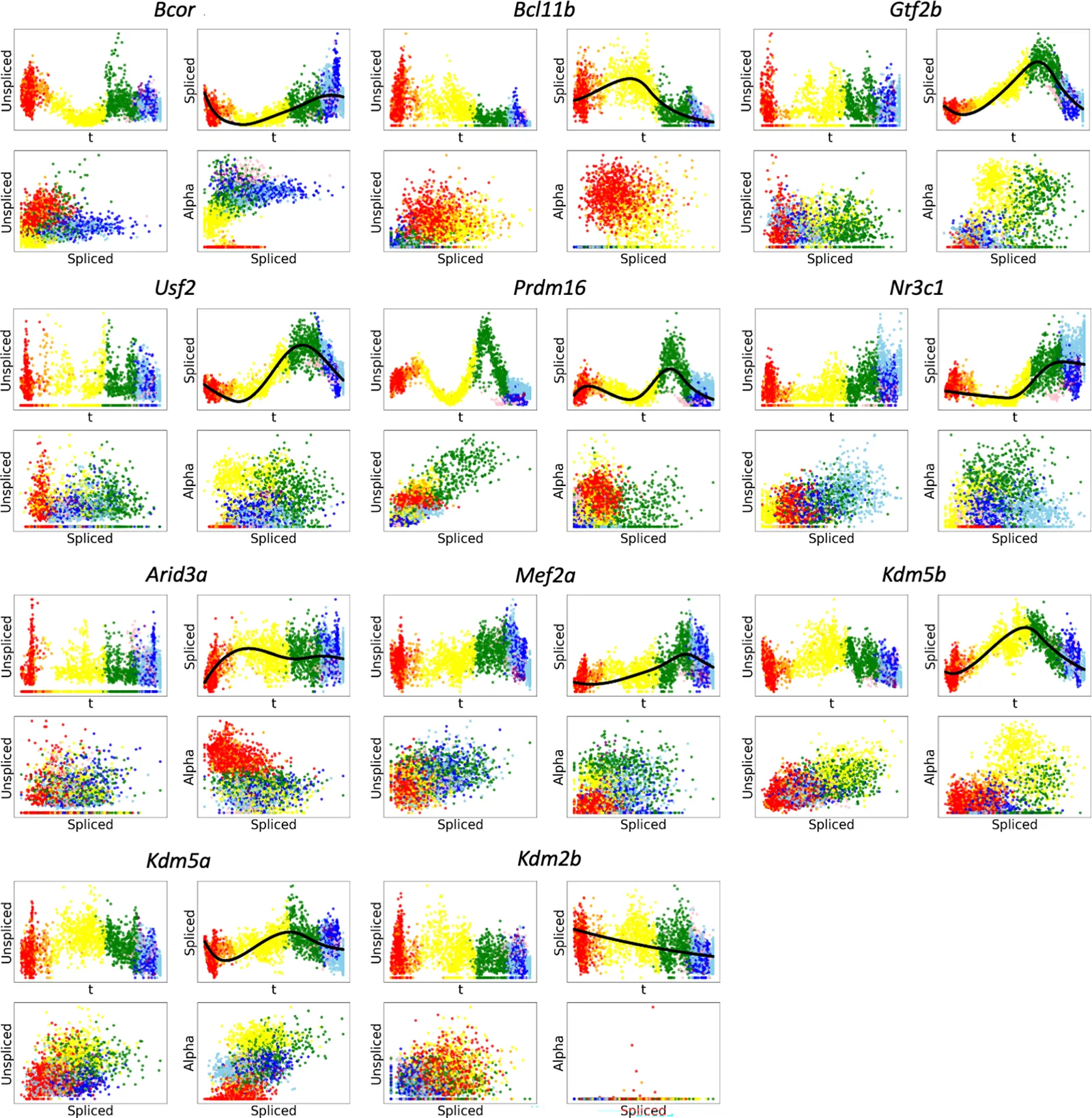
Dynamics fitting for additional transcription factors (TFs) on the pancreas dataset. TSvelo directly models the dynamic between transcription and spliced abundance on these genes. For each gene, four plots are displayed in a 2 × 2 layout: the u–t, s–t, u–s, and alpha–u plots.

In the conventional 2D unspliced–spliced phase portrait, cells from different clusters often overlap, limiting separability. By introducing the latent variable α, the representation is extended to a 3D space, which helps disentangle these mixed states and reveals a clearer phase structure. As shown in Figure 2c, the 3D representation achieves consistently higher *k*-nearest neighbor (kNN) classification accuracy for cell-state separation than the 2D u–s embedding (one-sided Mann–Whitney *U* test, p = 4.37 × 10⁻¹⁰). Details are provided in **Methods**. These results indicate that the 3D phase portrait provides improved separation of cell states and a stronger foundation for modeling the underlying dynamics along cell trajectories.

Additional quantitative evaluation is provided in Figure 2—figure supplement 1. TSvelo achieves the highest median velocity consistency, which demonstrates that the high-dimensional velocity vectors learned by TSvelo are mostly coherent within neighbor cells. TSvelo also achieves the highest median in-cluster coherence and cross-boundary correctness, which validates that TSvelo best fits the differentiation process within these cell types according to the ground-truth annotation.

Next, we show the dynamics for individual genes and demonstrate how the TSvelo model aids gene dynamics analysis by incorporating both transcriptional and splicing information. *Maml3*, *Anxa4*, and *Gstz1* are selected as examples because their unspliced–spliced 2D phase portrait exhibits mixed or overlapping patterns that are difficult to model using conventional RNA velocity approaches. Figure 2d presents the results of the TSvelo model on these genes, and Figure 2e presents the results of baseline approaches. Many previous approaches assume that the unspliced–spliced phase portrait exhibits an almond-shaped distribution (Weiler et al., 2022), which may not actually hold in the data. For *Maml3*, while the unspliced–spliced distribution predominantly follows the almond-shaped pattern, some cells, specifically Alpha cells (blue) and Beta cells (light blue), overlap with *Ngn3* high EP cells (yellow), indicating that these cell types cannot be distinctly separated using only the unspliced–spliced 2D phase portrait. Nevertheless, TSvelo can infer transcription representation from the expression of multiple TFs, which enables the model to distinguish these cell types (Figure 2d, f). *Anxa4* shows higher expression in Ductal cells (in red) compared to *Ngn3* low EP cells (in orange), which means its expression pattern exhibits an initial decrease followed by an increase. Such dynamics are not easily captured in the conventional unspliced–spliced phase portrait used by previous approaches, as many baseline methods implicitly assume a decreasing–then–increasing expression pattern. By comparison, TSvelo can still fit such expression patterns by using additional information from the 3D phase portrait. The visualization of dynamics fitting on more genes is provided at Figure 2—figure supplement 2 and Figure 2—figure supplement 3.

### TSvelo can better predict RNA velocity on gastrulation erythroid dataset

**Figure 3.**
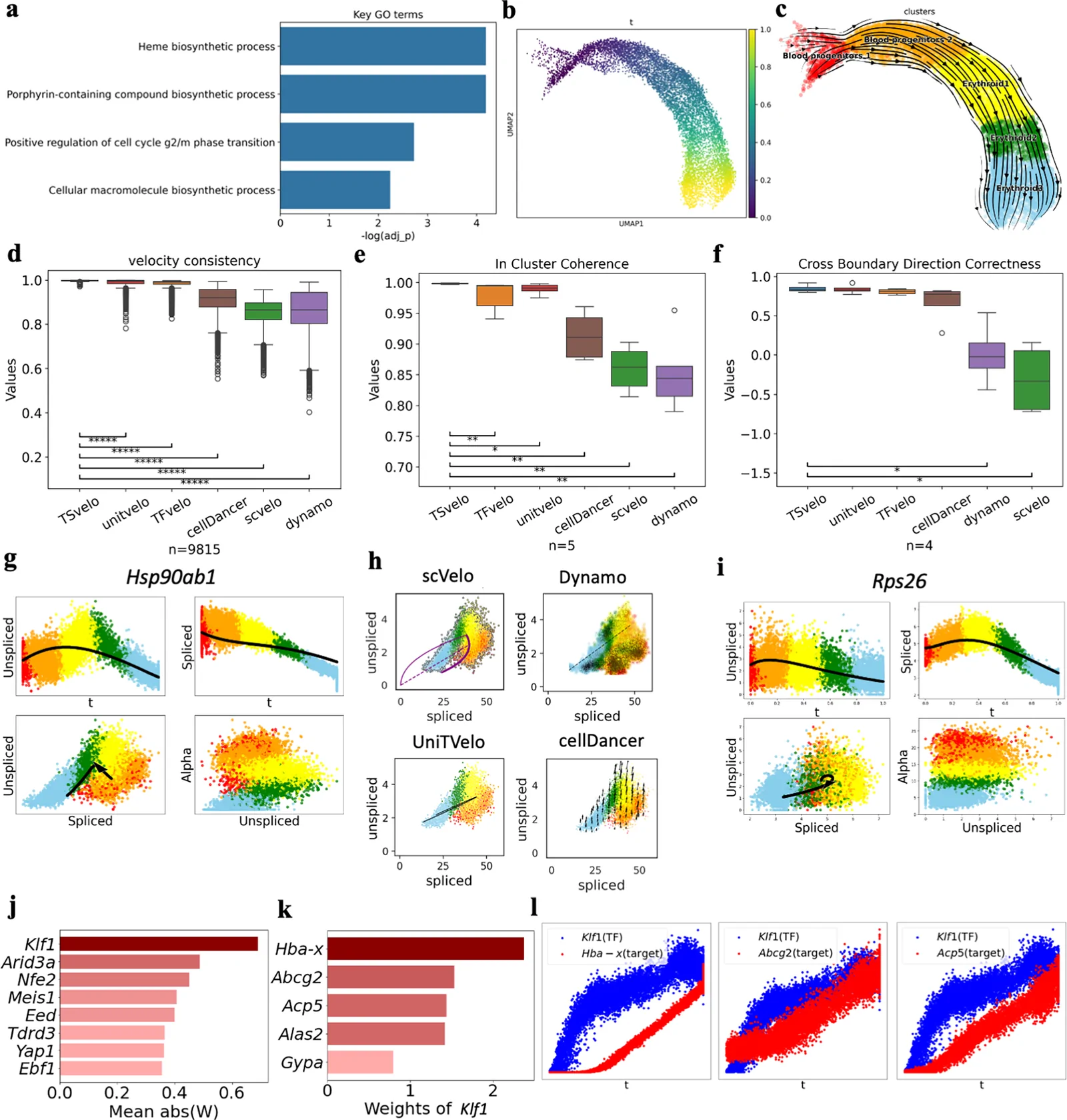
Results on gastrulation erythroid dataset. (**a**) The Gene Ontology (GO) terms which are mostly enriched in the selected velocity genes of TSvelo. (**b**) The pseudotime learned with TSvelo. (**c**) The stream plot for visualizing the RNA velocity inferred by TSvelo. The quantitative comparison between TSvelo and multiple baseline approaches in terms of velocity consistency (**d**), in-cluster coherence (**e**), and cross-boundary direction correctness (**f**). The down, central, and up hinges correspond to the first quartile, median value, and third quartile, respectively. The whiskers extend to 1.5× the interquartile range of the distribution from the hinge. 9815 samples are included in the boxplot for each method. (**g**) The dynamics fitting on *Hsp90ab1* obtained by TSvelo. Four plots are displayed in a 2 × 2 layout: the u–t, s–t, u–s, and alpha–u plots, where u, a, and alpha mean the abundance of unspliced mRNA, the abundance of spliced mRNA, and the learned transcriptional representation, respectively. (**h**) The phase portrait fitting on *Hsp90ab1* obtained by baseline approaches. (**i**) The dynamics fitting on *Rps26* obtained by TSvelo. (**j**) The transcription factors (TFs) with the highest ranked weights as identified by TSvelo. (**k**) Weights for *Klf1*’s targets, with the highest absolute weight. (**l**) Temporal dynamics of *Klf1* and its target genes with the highest weights along pseudotime, which includes *Hba-x, Alas2*, and *Gypa*. Figure 3—source data 1.The comparison of velocity consistency across different methods. Figure 3—source data 2.The comparison of in-cluster coherence across different methods. Figure 3—source data 3.The comparison of cross-boundary direction correctness across different methods.

We applied TSvelo to the gastrulation erythroid dataset, which is derived from the transcriptional profile of mouse embryos (Pijuan-Sala et al., 2019) and has been used in previous RNA velocity studies. This dataset primarily describes the differentiation process from blood progenitors to erythroid cells. First, using Gene Ontology (GO) term enrichment analysis (Figure 3a), we find that the velocity genes selected through TSvelo’s preprocessing strategies are mostly enriched in the erythropoiesis-related process, providing a preliminary validation of the biological plausibility of the inferred velocities.

**Figure 3—figure supplement 1.**
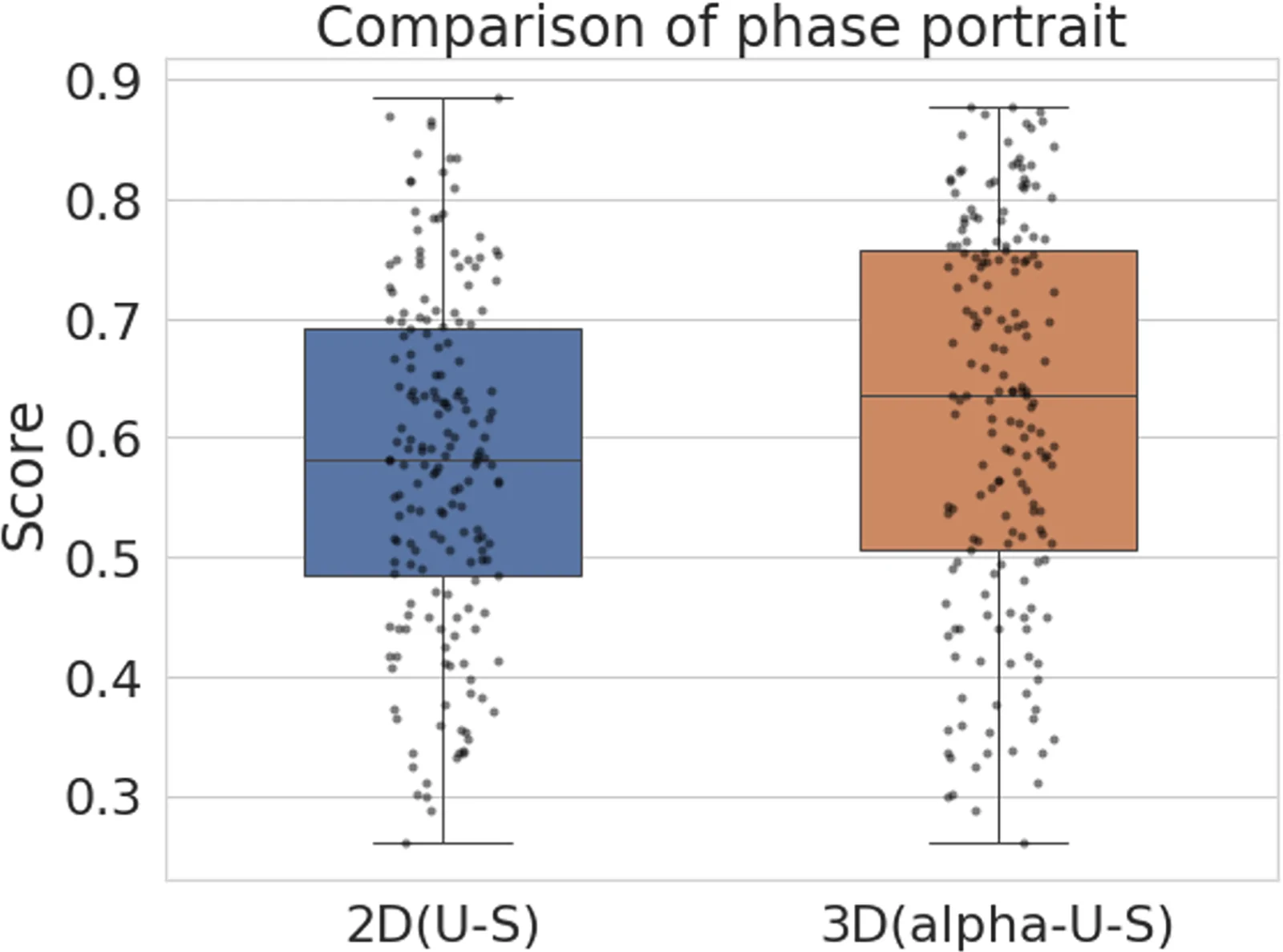
The evaluation of the separability of cell-type labels in both the 2D (unspliced–spliced) phase portrait and the 3D (α–unspliced–spliced) phase portrait for the gastrulation erythroid dataset.

**Figure 3—figure supplement 2.**
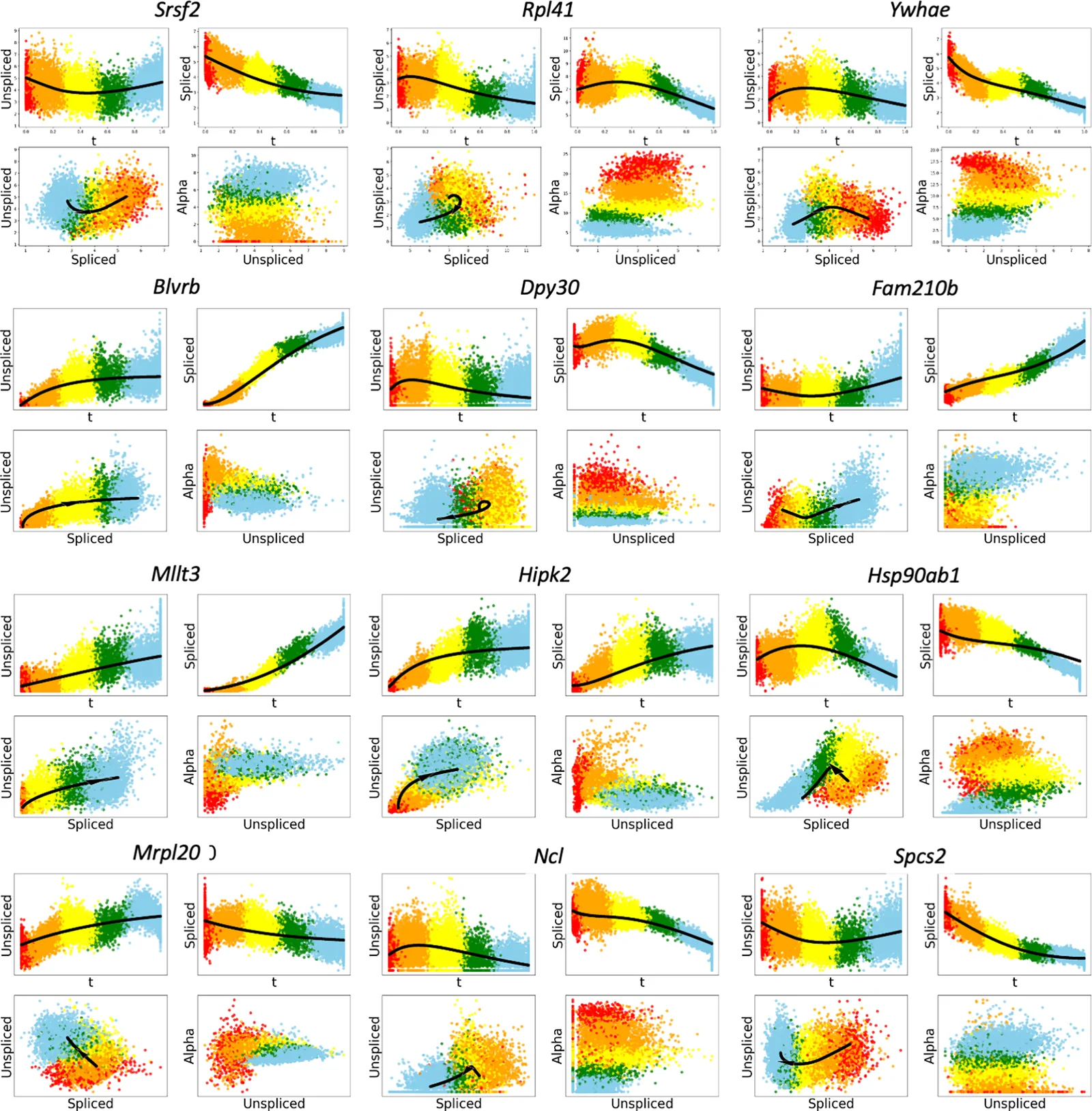
Dynamics fitting of velocity genes on the gastrulation erythroid dataset. For each gene, four plots are displayed in a 2 × 2 layout: the u–t, s–t, u–s, and alpha–u plots.

After applying TSvelo, both the inferred pseudotime (Figure 3b) and the velocity stream plot (Figure 3c) are compatible with current biological knowledge about the cell differentiation process. We also compare TSvelo with previous approaches. The results, shown in Figure 3d–f, demonstrate that TSvelo achieves the highest velocity consistency, the highest in-cluster coherence, and the highest cross-boundary correctness, showing that the high-dimensional dynamics predicted by TSvelo best capture the underlying biological processes.

We next analyze TSvelo modeling at the gene level in Figure 3g–i. The 3D phase portrait again provides a better basis for dynamic fitting, as it more effectively separates cells from different clusters than the traditional 2D phase portrait (Figure 3—figure supplement 1). For genes exhibiting higher noise or more complex patterns, TSvelo demonstrates its robustness in dynamic fitting by integrating both transcriptional and splicing signals. For *Hsp90ab1*, which exhibits a counter-clockwise pattern in the unspliced–spliced phase portrait (Figure 3g), in contrast to the clockwise dynamics typically assumed by most baseline approaches, it is difficult for previous methods to capture this behavior (Figure 3h), whereas TSvelo can still faithfully model such patterns. For genes such as *Rps26*, which have critical roles in the development of blood progenitors to erythroid (Liu and Karlsson, 2024), the unspliced–spliced data is so noisy that cells of different types overlap in phase portrait. TSvelo can still capture the gene dynamics and reveals differences in transcription rates (Alpha) across cell types (Figure 3i). In contrast, methods that rely solely on unspliced–spliced data from individual genes, such as scVelo, fail to accurately fit these dynamics and cannot identify them as velocity genes, which further validates TSvelo’s performance in complex scenarios. The visualization of dynamics fitting on more genes is provided at Figure 3—figure supplement 2.

From the TF–target weight matrices learned by TSvelo, we can extract key regulatory relationships. First, we calculate the mean absolute weight of each TF across its interactions with target genes. The TFs with the highest mean absolute weights are ranked and presented in Figure 3j. Notably, *Klf1*, a critical TF in blood progenitors (Mukherjee and Bieker, 2022), is assigned the highest weight. We then examine the target genes of KLF1, highlighting those with the highest weights (Figure 3k), among which *Hba-x* (Perseu et al., 2011), *Aals2* (Singleton et al., 2008), and *Gypa* Kuvardina et al., 2015 have been previously identified as key genes in erythroid differentiation and are known to be regulated by *Klf1*. A time-delay pattern is also observed between *Klf1* and its target genes (Figure 3l), indicating that *Klf1* expression increases first, followed by a corresponding upregulation of its target genes.

### TSvelo can capture gene dynamics well and predict cell fate on mouse brain data

We apply TSvelo to a 10x multi-omics dataset from the embryonic mouse brain, which includes both Assay for Transposase-Accessible Chromatin with sequencing (ATAC-seq) (Buenrostro et al., 2013) and scRNA-seq data. This dataset was previously used in the Multivelo study, which introduced an RNA velocity model designed for multi-omics datasets to capture the dynamics between chromatin accessibility and unspliced mRNA (Riba et al., 2022). As introduced by a previous study (Li et al., 2023), Radial Glia (RG) cells located in the outer subventricular zone give rise to neurons, astrocytes, and oligodendrocytes. During cortical development, neurons follow an inside-out layering pattern in which earlier-born neurons populate the deep cortical layers, whereas later-born neurons migrate past them to occupy more superficial layers (Nadarajah and Parnavelas, 2002). Additionally, RG cells are capable of producing intermediate progenitor cells (IPCs), which serve as neural stem cells and generate a variety of mature excitatory neurons within the cortical layers.

**Figure 4.**
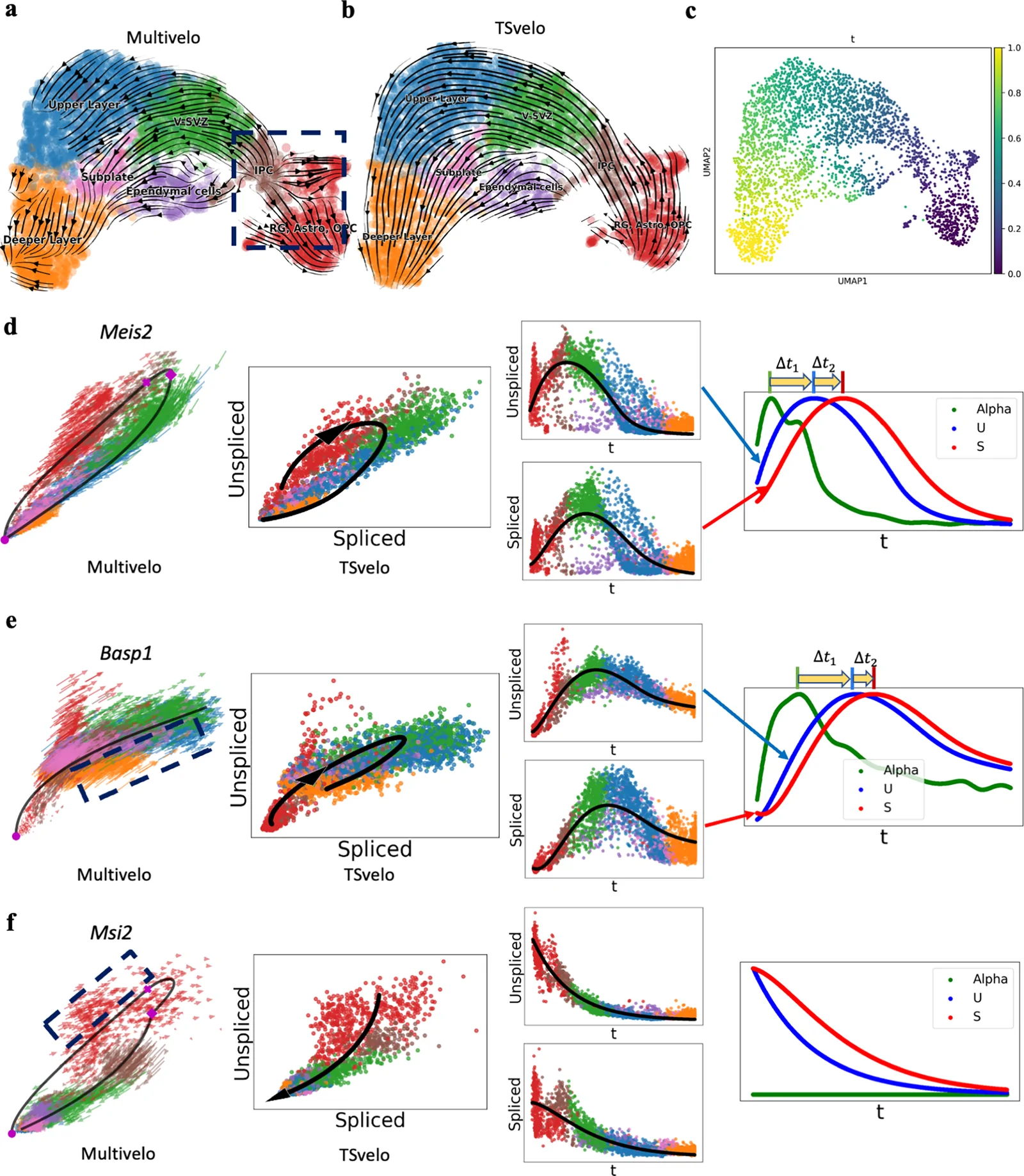
Results on mouse brain dataset. (**a**) The velocity stream inferred by Multivelo. (**b**) The stream plot for visualization of the RNA velocity inferred by TSvelo. (**c**) The pseudotime learned with TSvelo. The dynamics fitting on gene *Meis2* (**d**), *Basp1* (**e**), and *Msi2* (**f**) by both Multivelo and TSvelo. In each panel, the leftmost plot shows the phase portrait fitting of Multivelo, and the next two columns show TSvelo’s results. The rightmost plot shows the learned transcriptional rate (in green), unspliced abundance (in blue), and spliced abundance (in red) along the pseudotime. Since the transcriptional rate is calculated for each individual cell, we apply a Generalized Additive Model (GAM) to transcriptional representation across cells along the pseudotime and present GAM-fitting results to better visualize its trends in these plots.

Multivelo is chosen as the baseline in this study since it connects the regulation signal and RNA velocity in ATAC–unspliced–spliced space. TSvelo uses the same data processed by Multivelo for a fair comparison. The velocity stream obtained by TSvelo and Multivelo is shown in Figure 4a, b, respectively. TSvelo produces velocity patterns that appear more consistent with expected differentiation trends, especially for the RG (in red) to IPCs (in brown) process. The pseudotime inferred by TSvelo is also coherent with the whole cell development process (Figure 4c).

**Figure 4—figure supplement 1.**
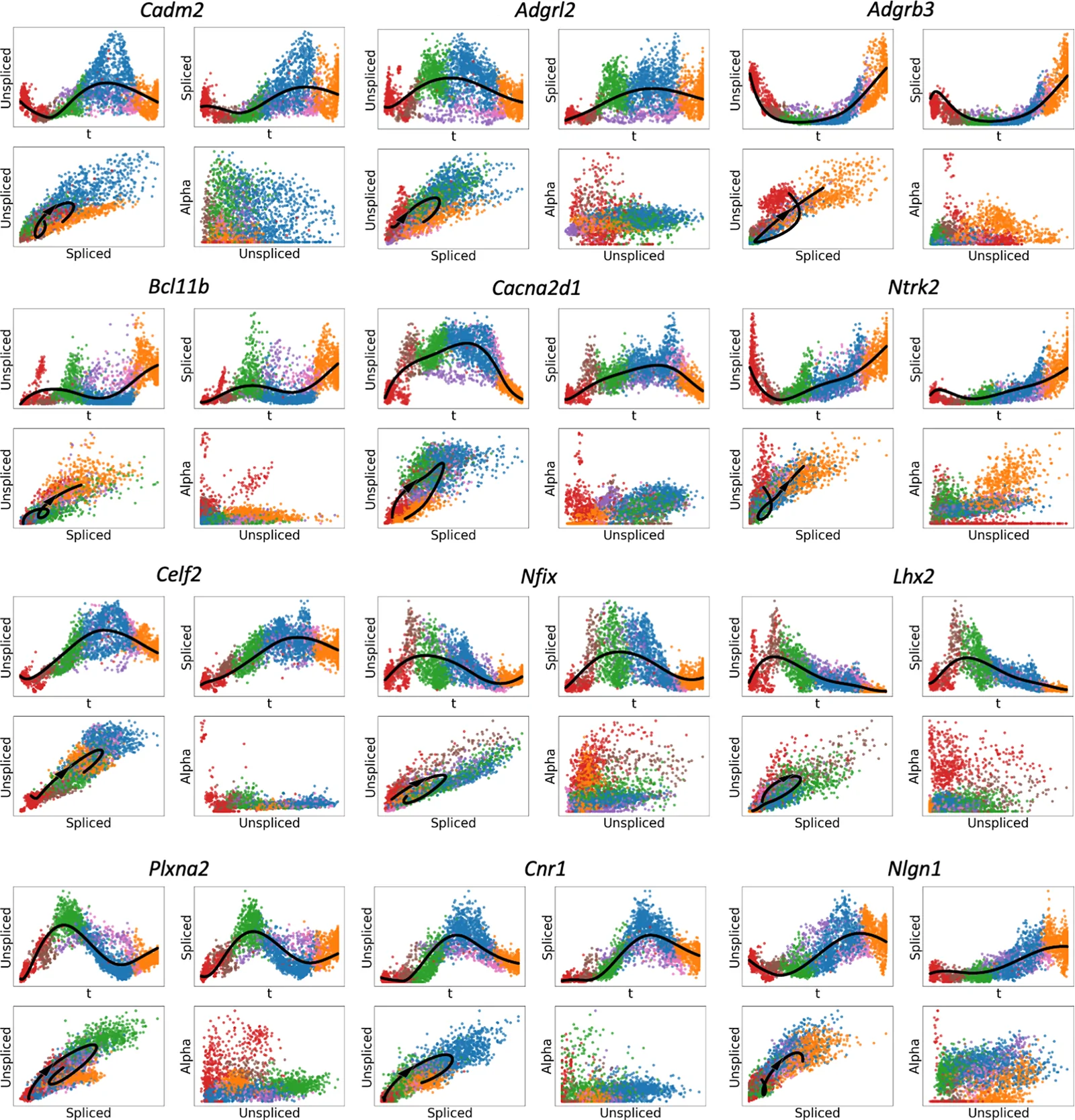
Dynamics fitting for additional genes on the mouse brain dataset. For each gene, four plots are displayed in a 2 × 2 layout: the u–t, s–t, u–s, and alpha–u plots.

**Figure 4—figure supplement 2.**
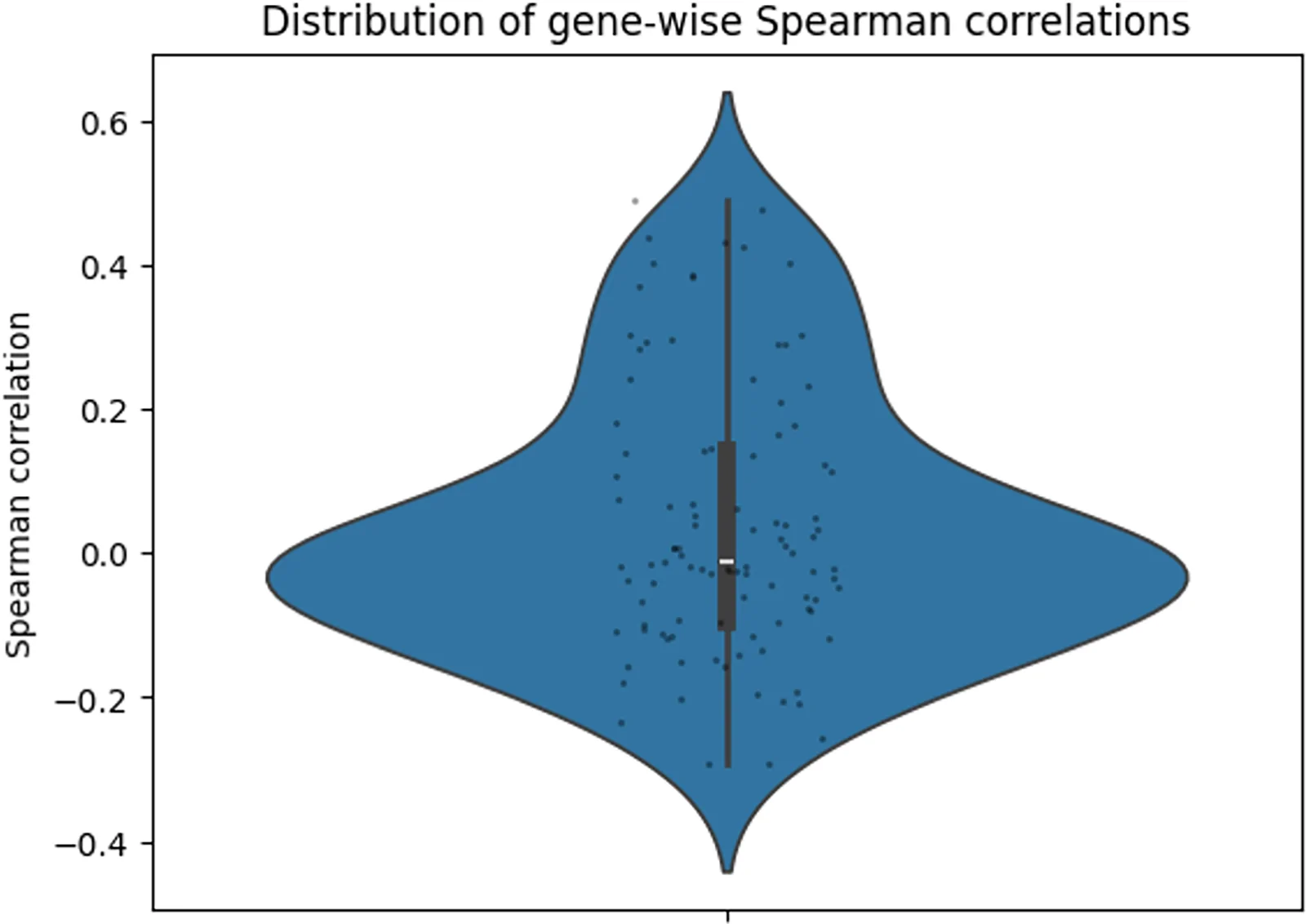
The distribution of gene-wise Spearman correlation between the estimated transcription rate in TSvelo and the chromatin accessibility rate in Multivelo.

**Figure 4—figure supplement 3.**
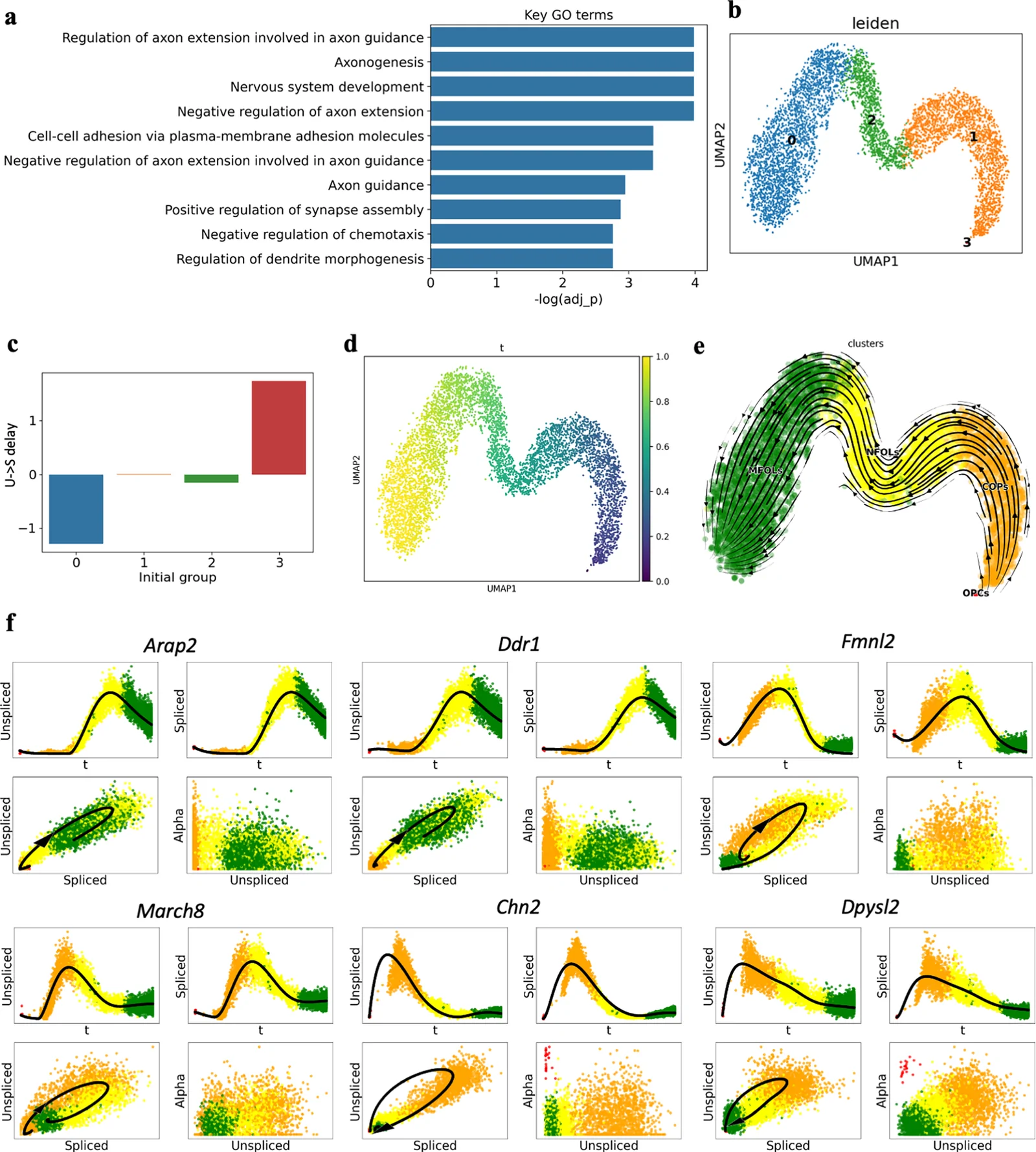
Results on pons dataset. (**a**) The Gene Ontology (GO) terms which are mostly enriched in the selected velocity genes of TSvelo. (**b**) The Leiden clustering. (**c**) The U-to-S delay under different initial cluster choices. (**d**) The pseudotime learned with TSvelo. (**e**) The stream plot for visualization of the RNA velocity inferred by TSvelo. (**f**) Dynamics fitting for multiple genes on the pons dataset. For each gene, four plots are displayed in a 2 × 2 layout: the u–t, s–t, u–s, and alpha–u plots.

We next further analyze the details in the phase portrait fitting. On *Meis2* (Figure 4d), both Multivelo and TSvelo successfully model its dynamics, which follows a clear pattern on the unspliced–spliced phase portrait. However, due to the high noise and sparsity inherent in ATAC-seq data, Multivelo encounters difficulty on genes that show significant overlap between different cell types in the unspliced–spliced phase portrait. For example, in the case of *Basp1* (Figure 4e), Multivelo incorrectly models their expression patterns as monotonically increasing, failing to capture the true dynamics, which involve initial upregulation followed by downregulation. In contrast, TSvelo’s prediction is consistent with known biological processes and identifies the delay from transcription to the unspliced mRNA and also the delay from unspliced to spliced mRNA (The rightmost plots in Figure 4e). *Msi2*, which has been reported to be highly expressed in neural stem/progenitor cells (Liu et al., 2019), is more accurately modeled by TSvelo, exhibiting a decreasing expression pattern during the differentiation process. By comparison, Multivelo fails to capture the correct trend at the initial stage (Figure 4f). We also show the learned transcriptional rate α as well as the predicted dynamics of unspliced and spliced RNA along pseudotime *t*, which clearly illustrate the time delays between transcription to unspliced and unspliced to spliced RNAs. The visualization of dynamics fitting on more genes is provided at Figure 4—figure supplement 1. Considering that ATAC- and TF RNA-based signals capture distinct aspects of gene regulation (Figure 4—figure supplement 2), integrating both modalities may be beneficial for future models that aim to more comprehensively characterize transcriptional regulation. Results on an additional mouse pons dataset are shown in Figure 4—figure supplement 3.

### TSvelo can predict cell fate and model lineage-specific gene dynamics for multi-lineage tasks

**Figure 5.**
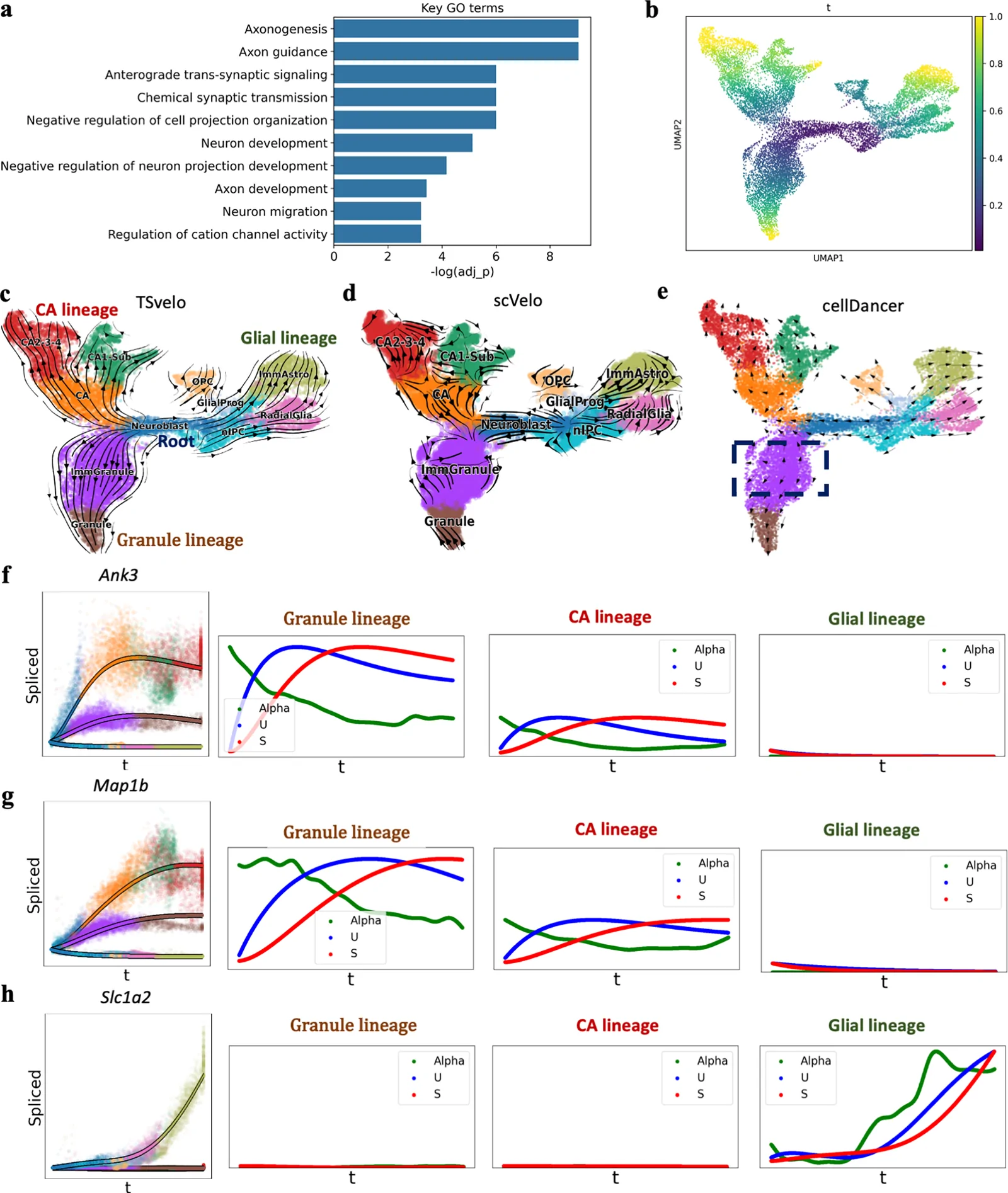
Results on the multi-lineage dentate gyrus dataset. (**a**) The Gene Ontology (GO) terms enriched in the selected velocity genes of TSvelo. (**b**) The pseudotime learned with TSvelo. (**c**) The stream plot for visualizing the RNA velocity inferred by TSvelo. Three lineages are detected, which are Granule lineage, CA lineage, and glial lineage. (**d**) The velocity stream inferred by scVelo. (**e**) The velocity stream inferred by cellDancer. The dynamics modeling of TSvelo on three axonogenesis-related genes, *Ank3* (**f**), *Map1b* (**g**), and *Slc1a2* (**h**). In the leftmost plot of each panel, the lines represent the predicted spliced abundance across all lineages, with the color indicating the cell types most associated with each pseudotime point along the corresponding lineage. Additionally, expression data for each lineage are shown as translucent points. The remaining plots in each panel display the dynamics of the learned transcriptional rate (in green), unspliced abundance (in blue), and spliced abundance (in red) along pseudotime for each lineage. The transcriptional representation in these plots is also processed using GAM fitting.

Given that multi-lineage differentiation is a common phenomenon in larger and larger scRNA-seq datasets, developing the RNA velocity model that can handle such complexity is essential. Because TSvelo demonstrates robust performance across various tasks, its applicability can be extended to more complex situations, such as scRNA-seq datasets where cells differentiate into multiple fates. We apply TSvelo to a multi-lineage dentate gyrus scRNA-seq dataset, which captures the differentiation process from neural blast cells to various cell types (Hochgerner et al., 2018). During preprocessing, the velocity genes selected by TSvelo are enriched in GO terms related to neural development, such as axonogenesis (GO:0007409), axon guidance (GO:0007411), and axon development (GO:0061564) (Figure 5a), which aligns with the biological processes described by the dataset.

**Figure 5—figure supplement 1.**
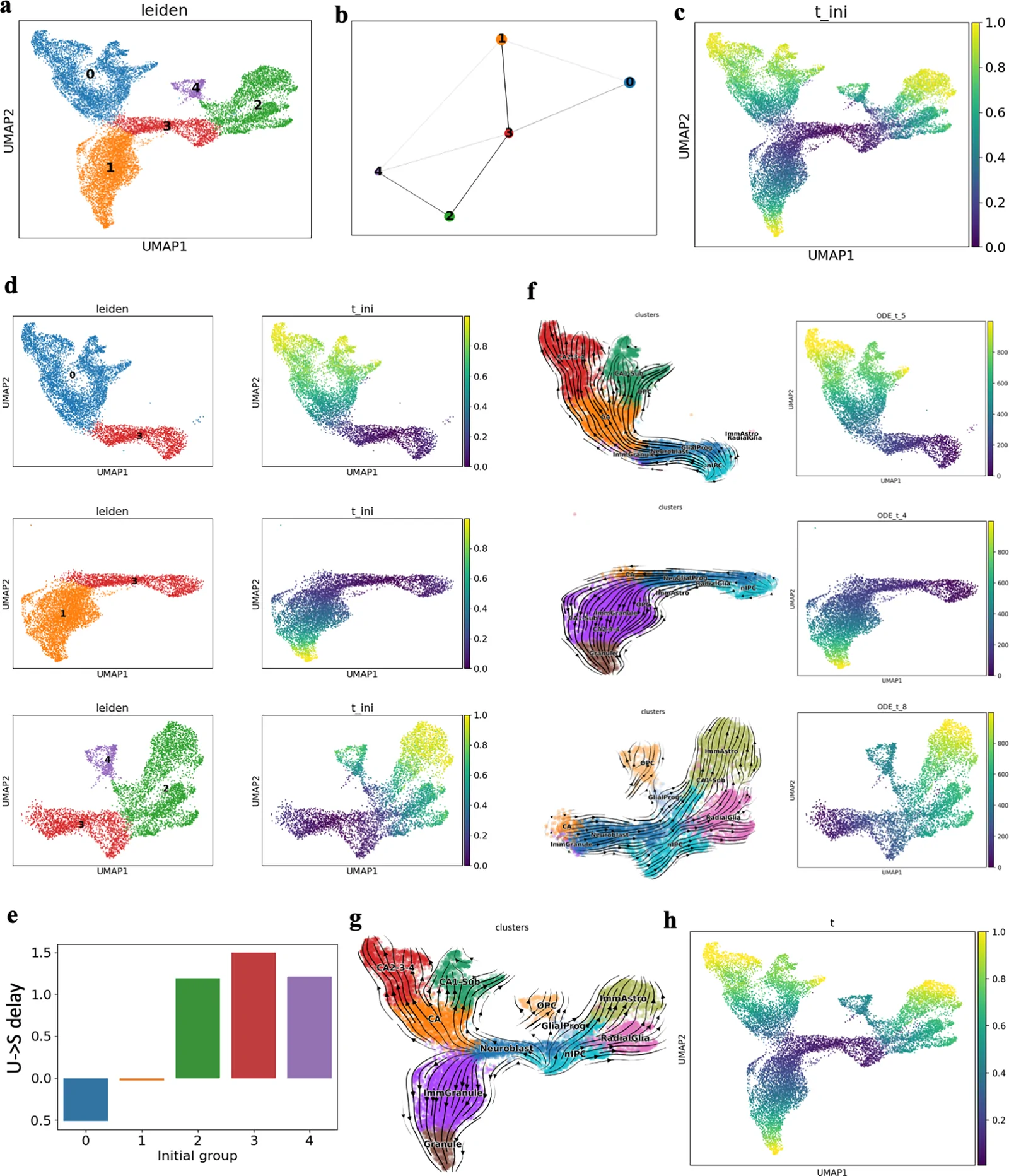
Lineages segmentation on the dentate gyrus dataset. (**a**) The Leiden clustering. (**b**) The cluster–cluster graph constructed with PAGA. (**c**) The diffusion pseudotime when choosing initial cluster 3 as the initial one. (**d**) The three branches detected when choosing initial cluster 3 as the initial one. (**e**) U-to-S delay under different initial cluster choices. (**f**) The velocity stream and pseudotime inferred by TSvelo on each lineage. (**g**) The velocity stream obtained with the combined velocities from each lineage. (**h**) The combined TSvelo pseudotime from each lineage.

**Figure 5—figure supplement 2.**
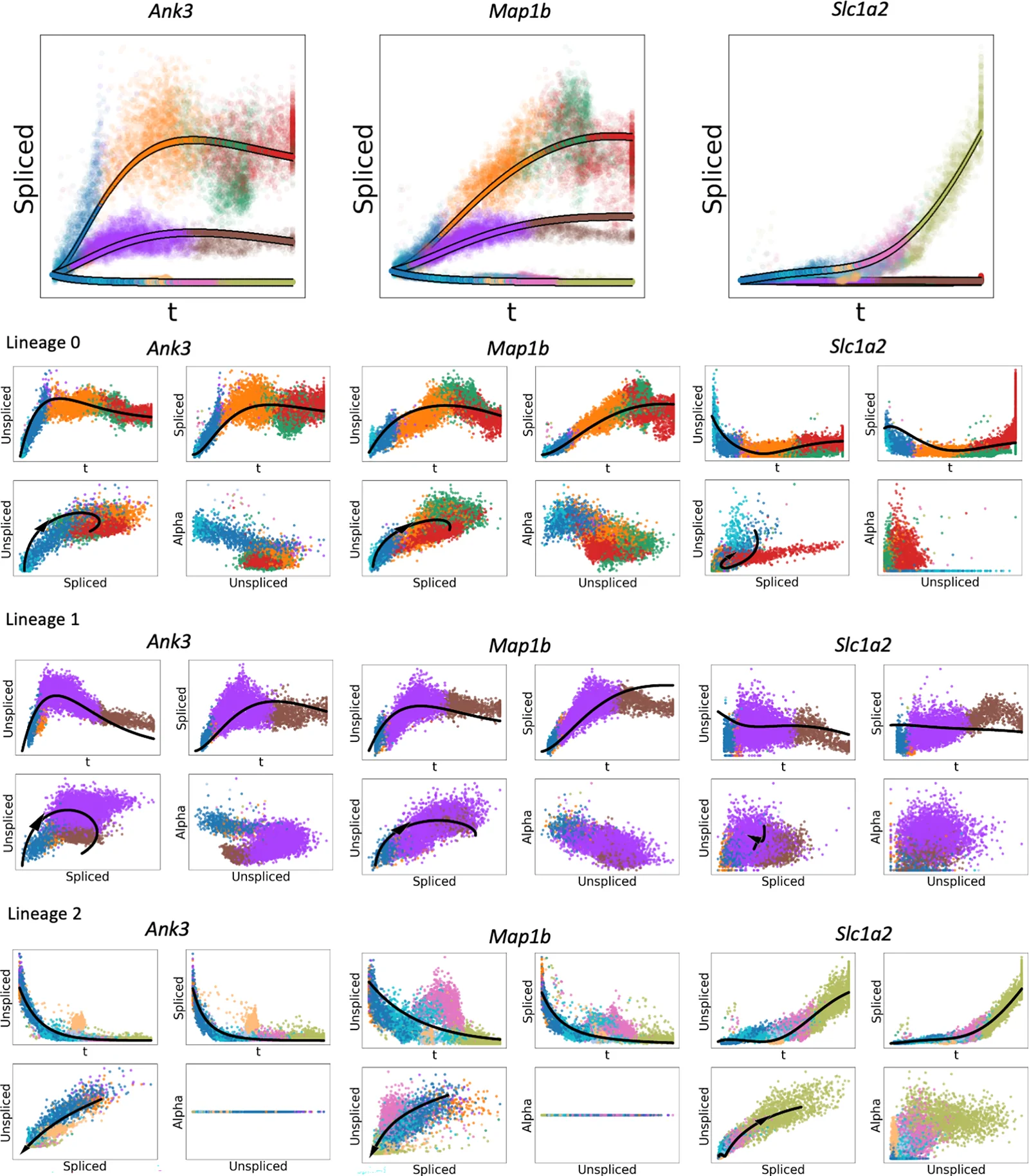
Dynamics fitting for genes *Ank3*, *Map1b*, and *Slc1a2* on each lineage of the dentate gyrus dataset. For each gene, four plots are displayed in a 2 × 2 layout: the u–t, s–t, u–s, and alpha–u plots.

TSvelo could correctly identify three lineages from this data. The lineage segmentation process is fully automated and does not require prior knowledge about the presence of multiple branches (details are provided at **Methods** and Figure 5—figure supplement 1). After applying the TSvelo model to each lineage and combining the results, the inferred pseudotime and velocity stream plots (Figure 5b, c) align with the ground-truth differentiation trajectory well, which begins with neural blast cells. We also compare TSvelo to baseline methods, including scVelo and cellDancer. Notably, cellDancer is designed to handle such multi-lineage data by modeling cell- and gene-specific transcriptional rates, splicing rates, and degradation rates using neural networks. As shown in Figure 5d, e, scVelo struggles with this multi-lineage dataset, and cellDancer fails to correctly capture the trajectory of immature granule cells (colored in purple).

Next, we analyze gene expression patterns across different lineages. TSvelo provides inferred dynamics along each lineage, revealing that the expression of *Ank3* in the granule and Cornu Ammonis (CA) lineages follows an increasing and then decreasing pattern. In contrast, the expression of *Ank3* in the glial lineage decreases to zero (Figure 5f). The similar pattern is also observed for other genes, such as *Map1b* (Figure 5g). Both *Ank3* (Leussis et al., 2012) and *Map1b* (Meixner et al., 2000) are associated with GO terms axonogenesis and axon guidance. This observation is consistent with the fact that the granule and CA (Douglas, 1967) predominantly consist of neurons, where axons are a critical component. In contrast, glial cells, such as astrocytes, typically lack axons, providing a potential explanation for these expression patterns. *Slc1a2* exhibits a distinct expression pattern, showing a significant increase in the glial lineage and a decrease in the granule and CA lineages. This pattern aligns with the observation that astrocytes are the primary cell type expressing *Slc1a2* (Sun et al., 2023). Details about dynamics fitting for genes on each lineage are provided at Figure 5—figure supplement 2.

### TSvelo can predict cell fate and model lineage-specific gene dynamics on the LARRY dataset

The Lineage and RNA Recovery (LARRY) method utilizes barcoded hematopoietic cells to trace both cell lineage and gene expression over time. LARRY has been successfully employed to track the in vitro differentiation of human blood cells, accurately capturing lineage trajectories and cell fates (Weinreb et al., 2020).

**Figure 6.**
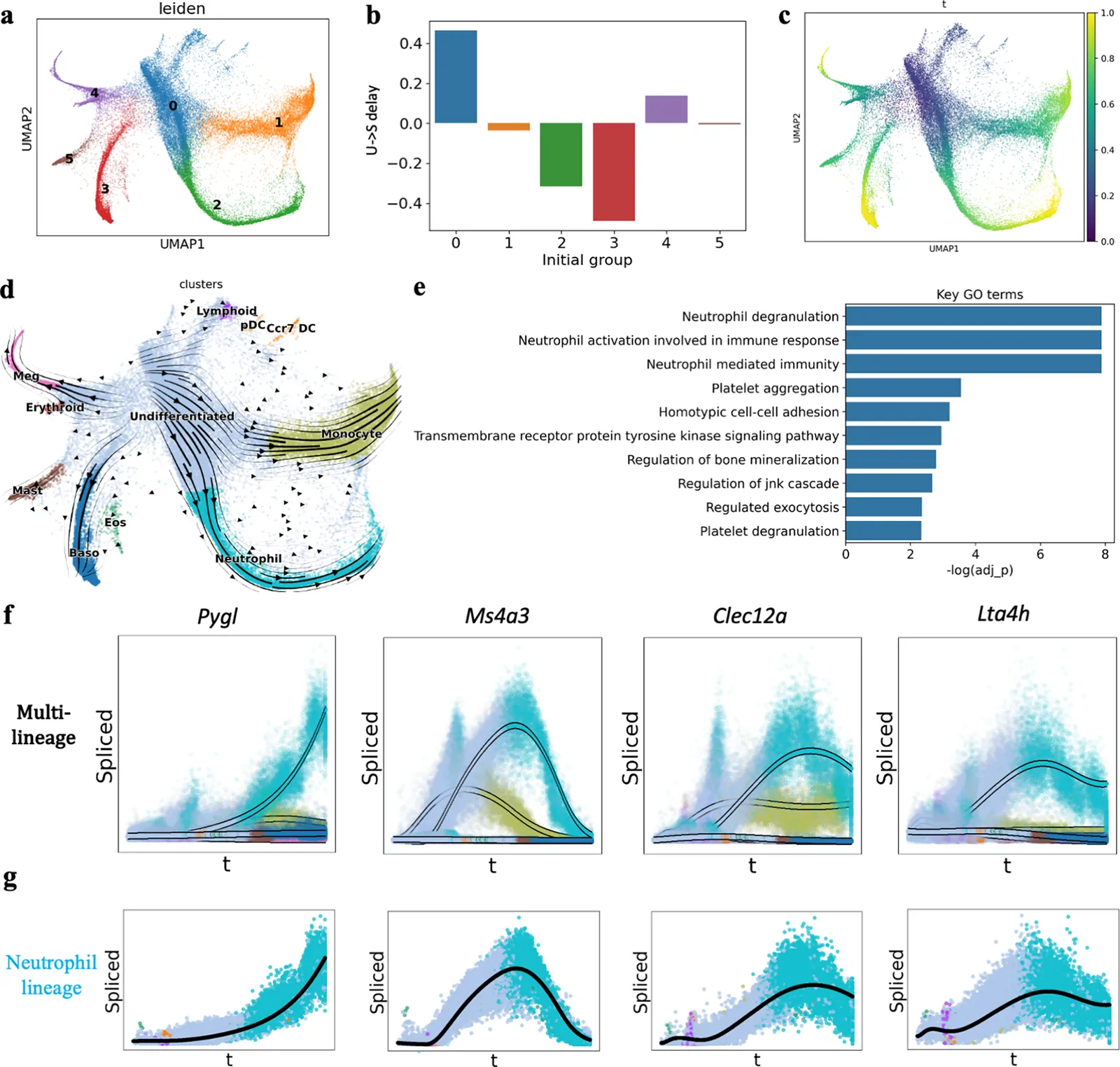
Results on the LARRY dataset. (**a**) The Leiden clustering on LARRY. (**b**) The initial Leiden cluster detection in preprocessing. (**c**) The pseudotime learned with TSvelo. (**d**) The stream plot for visualizing the RNA velocity inferred by TSvelo. (**e**) The Gene Ontology (GO) terms enriched in the selected velocity genes of TSvelo. (**f**) The dynamics modeling of TSvelo on four genes related to neutrophil development, which are *Pygl*, *Ms4a3*, *Clec12a*, and *Lta4h*. The lines represent the predicted spliced abundance across all lineages, with the color indicating the cell types most strongly associated with each pseudotime point along the corresponding lineage. (**g**) The dynamics modeling of *Pygl*, *Ms4a3*, *Clec12a*, and *Lta4h* on the neutrophil lineage.

On this LARRY dataset, which encompasses a total of 49,302 cells on multiple lineages, TSvelo effectively detects the initial Leiden clusters based on the unspliced-to-spliced delay (Figure 6a, b) and separates its lineages (details are provided at the **Lineage segmentation and pseudotime initialization** section in **Methods**). Furthermore, Figure 6c, d shows that the pseudotime and velocity stream inferred by TSvelo can capture the differentiation process, progressing from undifferentiated cells to distinct cell fates. The velocity genes selected by TSvelo are significantly enriched in processes related to neutrophil biology, such as neutrophil degranulation (GO:0043312), neutrophil activation in immune response (GO:0002283), and neutrophil-mediated immunity (GO:0002446).

We further analyzed the expression patterns of genes associated with neutrophil degranulation, focusing specifically on the neutrophil lineage. Using four representative genes as examples (Figure 6f, g), TSvelo can help observe the significant variation in the expression patterns across these genes. *Pygl*, which has been widely reported as a key gene in neutrophil (Borella et al., 2022), is almost exclusively expressed in neutrophil cells and exhibits an increasing expression pattern during neutrophil differentiation. In contrast, *Ms4a3* was examined across all lineages, and using TSvelo, we observed that its expression initially increases and then decreases in both the neutrophil and monocyte lineages. This pattern is consistent with prior studies identifying *Ms4a3* as a gene specifically expressed by granulocyte–monocyte progenitors (Liu et al., 2019). Similarly, *Clec12a*, another gene associated with neutrophil degranulation, shows a comparable expression trajectory during neutrophil differentiation. Previous research has indicated that *Clec12a* expression is highest in granulocyte–macrophage progenitors (Bill et al., 2018). *Lta4h* exhibits a pattern similar to that of *Clec12a*, suggesting it may play a significant role in the early stages of neutrophil differentiation. These findings further confirm the utility of TSvelo for gene-level analysis in multi-lineage differentiation datasets.

## Discussion

RNA velocity, utilizing unspliced/spliced data, has become a widely adopted concept for predicting cell fate and modeling gene dynamics. While several RNA velocity models have been proposed, most of them are based on phase portrait fitting in the unspliced–spliced space. However, due to the limited information and high noise inherent in the splicing data of individual genes, the short time delays between unspliced and spliced abundance, and the challenges posed by large-scale datasets with complex processes, most genes cannot be accurately modeled by previous RNA velocity methods. This limitation reduces the reliability and robustness of downstream analyses.

Gene expression is a complex biological process within cells, involving multiple regulatory mechanisms from DNA to the matured RNA. In this process, transcription and splicing are two crucial steps to determine the final gene expression. Due to the mathematical complexity, previous methods cannot jointly model gene regulation, transcription, and splicing. TSvelo comprehensively models the cascade of the whole process using an interpretable ODE framework, allowing for learning dynamics of all velocity genes simultaneously. TSvelo could accurately model gene dynamics, predict cell fate, detect the key regulatory relations, and handle multi-lineage datasets. Results on six scRNA-seq datasets demonstrate that TSvelo is a valuable approach for RNA velocity modeling.

One limitation of TSvelo is its reliance on predefined TF–target regulatory priors, which may be incomplete and may not fully capture context-specific regulatory relationships in primary tissues or at single-cell resolution. Second, jointly modeling high-dimensional regulatory interactions and RNA kinetics introduces additional computational overhead compared with some existing approaches, as reflected in runtime and memory analyses (Appendix 1—tables 2 and 3, and Figure 1—figure supplement 6). Third, accurately capturing cell-cycle transitions remains challenging, since cyclic gene-expression programs may violate the assumption of unidirectional state progression and thereby complicate velocity-based trajectory inference. Finally, like many RNA velocity and trajectory inference methods, TSvelo assumes that the input data reflects underlying dynamic biological processes, highlighting the importance of appropriate preprocessing checks when applying such methods to non-dynamic datasets (Figure 1—figure supplement 7).

Looking forward, there are several opportunities to further strengthen the framework. Incorporating context-specific regulatory information, such as single-cell chromatin accessibility data, may improve transcriptional modeling. Moreover, TSvelo currently assumes constant gene-specific splicing and degradation rates. Since splicing factors also play significant roles in regulating other genes during the splicing process (Rogalska et al., 2024), and there are still complex mechanisms of mRNA degradation (Shyu et al., 2008), further exploration could enhance velocity’s modeling and bring new biological insights in the future.

## Methods

**Key resources table:** 

Reagent type (species) or resource	Designation	Source or reference	Identifiers	Additional information
Software, algorithm	TSvelo	https://github.com/lijc0804/TSvelo, copy archived at Li, 2026		

### Preprocessing for scRNA-seq data

The unspliced and spliced RNA abundances are preprocessed with multiple steps, which include highly variable genes (HVGs) selection, normalization, log transformation, kNN smoothing, and clustering. Following the data preprocessing procedures outlined in scVelo (Bergen et al., 2020), we first select the top 2000 HVGs and normalize their expression profiles by dividing by the total counts in each cell. A nearest-neighbor graph (with 30 neighbors by default) was constructed based on Euclidean distances in principal component analysis space (with 30 principal components by default) on log-transformed gene expression data. Subsequently, we compute the first- and second-order moments (mean and uncentered variance) for each cell across its nearest neighbors. These steps are performed by using scvelo.pp.filter_and_normalize() and scvelo.pp.moments().

### Acquiring prior knowledge of gene regulatory relations

To construct a prior gene network for the selected HVGs, we utilize TF–target annotations from the ENCODE (Feingold, 2004) and ChEA (Lachmann et al., 2010) databases. If a regulatory interaction between a TF and its target gene is identified in either of these two databases, the TF is included in the set of regulators for modeling the dynamic behavior of the target gene. During model optimization, the contribution of each prior regulatory edge is adaptively adjusted, allowing unsupported interactions to be effectively down-weighted. As a result, TSvelo is expected to be relatively robust to false-positive TF–target annotations. By contrast, missing true regulatory interactions are not represented in the candidate regulator set and therefore cannot contribute to the learned regulatory dynamics, making the model potentially more sensitive to false negatives in the prior network. Ablation studies on the selection of TF–target resources (Figure 1—figure supplements 4 and 5) verify that integrating multiple TF–target resources can improve performance by increasing regulatory coverage and reducing false negatives in the prior network.

### Velocity genes selection

Previous studies Bergen et al., 2020 have demonstrated that only a subset of genes, termed ‘velocity genes’, can be accurately fitted in the unspliced–spliced phase portrait by RNA velocity models. Inspired by the notion that velocity genes provide high-quality data for dynamic modeling in the phase portrait, we propose to select velocity genes during the preprocessing step. The velocity genes are selected based on the premise that they exhibit clear dynamics in the unspliced–spliced phase portrait, which is necessary for precise modeling of splicing dynamics. The selection is based on the similarity between cell–cell neighborhood relationships in UMAP space and those observed in gene-specific unspliced–spliced phase portraits. In detail, we first compute the cell–cell neighborhood graph, *Graph*, using scvelo.pp.neighbors() on all HVGs. Next, we construct an anndata object *adata*_*g*_ for each gene, which contains only the unspliced and spliced expression data of that gene. We then apply scvelo.pp.neighbors() to each *adata*_*g*_ to calculate the gene-specific neighborhood graph, denoted as *Graph*_*g*_. Subsequently, we compute the similarity between *Graph* and each *Graph*_*g*_. The top 100 genes with the highest similarity are selected as velocity genes. These genes are characterized by phase portraits that exhibit a structure similar to that of the UMAP space, thereby enhancing the separation of cells from different types. As a result, the splicing dynamics of these velocity genes are more likely to be captured by RNA velocity models.

### Lineages segmentation and pseudotime initialization

Based on the normalized gene expression data, we perform Leiden clustering using scanpy.tl.leiden() with a low resolution (default resolution = 0.1). These Leiden clusters are utilized to identify lineages and determine the differentiation direction for each lineage. Subsequently, we apply PAGA (scanpy.tl.paga()) to assess the connectivity between Leiden groups.

By setting one Leiden group as the initial state, we infer pseudotime using diffusion pseudotime (DPT) with scanpy.tl.dpt(). To detect lineages, we start from the selected initial Leiden cluster and filter the PAGA graph by applying a threshold (default = 0.02). Edges with weights below this threshold are removed, leaving only the shortest path from the initial state to each group. The remaining paths are considered as the detected lineages.

Given a lineage, TSvelo could detect the orientation of differentiation along it, which is based on the fact that unspliced RNA precedes spliced RNA in the expression pattern (Ge et al., 2025). For each gene, TSvelo computes the Spearman correlation between unspliced and spliced abundance across different moving steps along the DPT. The time number of moving steps along unspliced to spliced expression, which maximizes the Spearman correlation, is considered the U-to-S delay (Figure 1a shows an example of U-to-S delay with gene EML5). The U-to-S delay for each lineage is calculated as the mean U-to-S delay for all genes on it. And the average U-to-S delay across all lineages provides the overall U-to-S delay for the initial Leiden cluster. Finally, the initial Leiden cluster with the highest U-to-S delay is considered the correct initiation. The DPT under this condition is used to initialize pseudotime for TSvelo. If multiple lineages are detected, TSvelo will model each lineage independently and subsequently merge them in the final step. A detailed explanation with an example illustrating how lineages are segmented is provided in Figure 5—figure supplement 1. Results of the initialization strategy on all datasets are shown in Figure 1—figure supplement 3 demonstrating that clusters with the highest U-to-S delay scores can be reliably identified as the initial cell states. We also provide a simulation study to verify this initialization strategy in Figure 1—figure supplement 2.

### The ODE model in TSvelo

Suppose $u_{g}$ and $s_{g}$ are the abundance of unspliced and spliced RNA, and $\alpha _{g}\left (t\right),\beta _{g}$, and $\gamma _{g}$ are the transcription, splicing, and degradation rates, respectively. The dynamics of a velocity gene g is modeled as

(4) $$\displaystyle \frac{du_{g}\left (t\right)}{dt}=\alpha _{g}\left (t\right)- \beta _{g}u_{g}\left (t\right)$$

(5) $$\displaystyle \frac{ds_{g}\left (t\right)}{dt}=\beta _{g}u_{g}\left (t\right)- \gamma _{g}s_{g}\left (t\right)$$

We assume that the gene and cell-specific transcriptional rate $\alpha _{g}\left (t\right)$ is influenced by the expression of TFs, while $\beta _{g}$ and $\gamma _{g}$ are gene-specific constant parameters. Considering the wide usage of linear models for gene relations in previous studies (Li et al., 2024a; Tran et al., 2022; Song et al., 2023; Liu et al., 2011; Wang et al., 2023), we model $\alpha _{g}\left (t\right)$ as

(6) $$\displaystyle  \alpha _{g}\left (t\right)=ReLU\left (\sum _{i\in TFs\left (g\right)}w_{gi}\ast s_{i}\left (t\right)\right)$$

In order to include more TFs in the modeling, we reserve those TFs that are not selected as velocity genes. These TFs are excluded from the velocity genes selection because their unspliced–spliced expression does not provide sufficient information, primarily due to high noise in the unspliced RNA. Consequently, we incorporate these TFs, whose spliced abundance is denoted as s`, into the TSvelo by directly modeling the process between transcriptional signal to mature RNA,

(7) $$\displaystyle \frac{ds'_{g}\left (t\right)}{dt}=\alpha _{g}\left (t\right)- \gamma _{g}s'_{g}\left (t\right)$$

As a result, we can get the ODE model with parameters matrix A:

(8) $$\displaystyle \begin{array}{c}\left [\begin{array}{c}dU/dt\\dS/dt\\dS^{'}/dt\end{array}\right ]=A\left [\begin{array}{c}U\\S\\S'\end{array}\right ]=\left [\begin{array}{ccc}- B & W & W'\\B & - \Gamma & 0\\0 & W' & W'- \Gamma '\end{array}\right ]\left [\begin{array}{c}U\\S\\S'\end{array}\right ]\end{array}$$

$U=[u_1,u_2,\dots,u_G]^T\in R^G$, $S=[s_1,s_2,\dots,s_G]^T\in R^G$, $S=[s_{G+1},s_{G+2},\dots,s_{G+K}]^T\in R^K$, G is the number of velocity genes, and K is the number of additional TFs which are not selected as velocity genes. $B \in R^{G\times G}$ and $\Gamma \in R^{G\times G}$ are the diagonal matrices consisting of splicing rates $\left [\beta _{1},\beta _{2}\ldots ,\beta _{G}\right ]$ and degradation rates $\left [\gamma _{1},\gamma _{2}\ldots ,\gamma _{G}\right ]$ for velocity genes, respectively. $\Gamma '\in R^{K\times K}$ is the diagonal matrix consisting of degradation rates $\left [\gamma _{G+1},\gamma _{G+2}\ldots ,\gamma _{G+K}\right ]$ for these additional TFs. $W\in R^{G\times G}$ and $W^{'}\in R^{K\times K}$ denote the matrices representing the TF–target relationships for the TFs selected as velocity genes and those not selected as velocity genes, respectively. Using these gene–gene weight matrices, TSvelo can model the gene- and cell-specific transcriptional rate $\alpha _{g}$. The whole parameters matrix $A\in R^{\left (2G+K\right)\times \left (2G+K\right)}$.

### Optimizing global time and Neural ODE in EM framework

In TSvelo, the dynamics described in Equation 7 are implemented using a Neural ODE model. Given an initial state and the parameters in matrix A, the Neural ODE model can compute the values of U and S at any time step. This approach eliminates the need for an analytical solution for unspliced and spliced expression, enhancing the flexibility of TSvelo. By default, the number of time steps in the ODE is set to 1000. The ReLU activation function is applied to the parameters $\alpha _{g}$, $\beta _{g}$, and $\gamma _{g}$, in order to prevent them from taking negative values. Notably, TSvelo does not incorporate deep neural networks or encoders. Since TSvelo models all genes simultaneously, we normalize the unspliced and spliced abundances of each gene by its standard deviation before inputting the data into the Neural ODE model to ensure that the influence of each gene is balanced.

TSvelo optimizes the pseudotime t and parameters matrix A in ODE iteratively using an EM approach. The maximum number of iterations is set to 30 by default, with an early stopping criterion applied. Given the parameters in the ODE system, we can compute the predicted values of unspliced (for velocity genes) and spliced (for velocity genes and additional TFs) at all time steps, denoted as $\hat {U}\left (t_{step};A\right)$, $\hat {S}\left (t_{step};A\right)$, and $\hat {S^{'}}\left (t_{step};A\right)$, where $t_{step}\in \left \{0,1,2\ldots ,999\right \}$. Given the time steps assignment $t_{c}$ for each cell c, we can get the expected values for velocity genes at all cells as $\hat {U}\left (t_{c};A\right),\hat {S}\left (t_{c};A\right)$, and $\hat {S^{'}}\left (t_{c};A\right)$. The loss function used in the EM algorithm is the mean squared error between the expected value and the observed data.

(9) $$\displaystyle  Loss=\frac{1}{c}\sum _{c}\left [\left (\hat {U}\left (t_{c};A\right)- U_{c}\right)^{2}+\left (\hat{S}\left (t_{c};A\right)- S_{c}\right)^{2}+\left (\hat {S^{'}}\left (t_{c};A\right)- S^{'}_{c}\right)^{2}\right ]$$

The EM algorithm will iteratively update the time assignment and parameters in the Neural ODE by minimizing the above loss function.

In the E-step, following the strategy adopted in the scVelo model (Bergen et al., 2020), TSvelo updates the time step assigned to each cell using grid search with time steps range $t_{step}\in \left \{0,1,2\ldots ,999\right \}$, which is achieved by finding the minimum distance between the observed unspliced–spliced expression and the model prediction ($\hat {U}$ and $\hat{S}$) at all time steps.

(10) $$\displaystyle  min_{t_{c}}\frac{1}{c}\sum _{c}\left [\left (\hat{U}\left (t_{c};A\right)- U_{c}\right)^{2}+\left (\hat{S}\left (t_{c};A\right)- S_{c}\right)^{2}+\left (\hat{S^{'}}\left (t_{c};A\right)- S^{'}_{c}\right)^{2}\right]$$

In the M-step, the parameter matrix A is updated using gradient descent within the Neural ODE model to minimize the loss function. Here we use the prior gene regulation knowledge to constrain the weight matrix W and $W^{'}$ in A. Only if gene i is annotated as a TF for gene j in the ENCODE or ChEA databases, the corresponding $w_{ij}$ could be learnable. Otherwise, $w_{ij}$ is fixed at zero. The usage of prior knowledge could keep the matrix sparse and avoid overfitting. The Neural ODE module is implemented using the torchdiffeq package and trained with the Adam optimizer, a learning rate of 0.05, and a maximum of 500 epochs, incorporating an early stopping criterion to prevent overfitting.

(11) $$\displaystyle  min_{A}\frac{1}{c}\sum _{c}\left [\left (\hat{U}\left (t_{c};A\right)- U_{c}\right)^{2}+\left (\hat{S}\left (t_{c};A\right)- S_{c}\right)^{2}+\left (\hat{S^{'}}\left (t_{c};A\right)- S^{'}_{c}\right)^{2}\right ]$$

By performing the EM optimization, TSvelo could fit the high-dimensional unspliced–spliced data across multiple genes well, and learn the global pseudotime for each cell. After the training procedure, the RNA velocity could be calculated using those parameters in matrix A, which could be further used for cell fate prediction.

### Metrics for evaluating

(1) Velocity consistency (VCon). We used the scvelo.velocity_confidence() function from scVelo to evaluate velocity consistency, interpreting the results as a measure of how consistent velocities are within neighboring cells. Velocity consistency is especially suitable for evaluating the RNA velocity modeling on single lineage. For each cell c, the velocity consistency is calculated as follows:

(12) $$\displaystyle  VCon\left (c\right)=\frac{1}{|\left \{c^{'}\in N\left (c\right)\right \}|}\sum _{c^{'}\in N\left (c\right)}\frac{\boldsymbol v_{c}\cdot \boldsymbol v_{c^{'}}}{| \boldsymbol v_{c}| \cdot |\boldsymbol v_{c^{'}}| }$$

where $N\left (c\right)$ represents the neighboring cells of a given cell c. $v_{c}$ and $v_{c^{'}}$ denote the low-dimensional velocity vectors of cell c and its neighboring cell $c^{'}$.

(2) Cross-boundary direction correctness (CBDir). Cross-boundary direction correctness is initially introduced by VeloAE (Qiao and Huang, 2021), which assesses the accuracy of transitions from a source cluster to a target cluster by examining the boundary cells, and requires ground-truth annotations. We directly run the function unitvelo.evaluate() provided in UniTVelo Gao et al., 2022 to obtain the Cross-boundary direction correctness. In detail, the CBDir is calculated as follows:

(13) $$\displaystyle  CBDir\left (c\right)=\frac{1}{|\left \{c^{'}\in C_{A}\cap N\left (c\right)\right \}|}\sum _{c^{'}\in C_{A}\cap N\left (c\right)}\frac{\boldsymbol v_{c}\cdot \left (\boldsymbol x_{c^{'}}-\boldsymbol x_{c}\right)}{|\boldsymbol v_{c}| \cdot |\boldsymbol x_{c^{'}}-\boldsymbol x_{c}| }$$

where $C_{A}$ denotes the set of cells in the target cluster *A*, and $N\left (c\right)$ represents the neighboring cells of a given cell c. $\boldsymbol v_{c}$ and $\boldsymbol x_{c}$ denote the low-dimensional velocity and state vectors of cell c, respectively, and $\boldsymbol x_{c^{'}}$ denotes the state vector of its neighboring cell.

(3) Within-cluster velocity coherence (ICCoh). Within-cluster velocity coherence is initially introduced by VeloAE (Qiao and Huang, 2021), which measures the coherence of velocities within a single cluster using a cosine similarity score between cell velocities. We applied the function unitvelo.evaluate() provided by UniTVelo (Gao et al., 2022) to directly compute the within-cluster velocity coherence. Using the same notation as defined above, the CBDir is calculated as follows:

(14) $$\displaystyle  ICCoh\left (c\right)=\frac{1}{|\left \{c^{'}\in C_{A}\cap N\left (c\right)\right \}|}\sum _{c^{'}\in C_{A}\cap N\left (c\right)}\frac{\boldsymbol v_{c}\cdot \boldsymbol v_{c^{'}}}{|\boldsymbol v_{c}| \cdot |\boldsymbol v_{c^{'}}|}$$

### The comparison between the 3D phase portrait and traditional 2D phase portrait

To quantitatively assess whether TSvelo can distinguish cell types, we evaluated the separability of cell-type labels in both the 2D (unspliced–spliced) phase portrait adopted by previous RNA velocity approaches, and the 3D (α–unspliced–spliced, α denotes the transcriptional rate) phase portrait introduced by TSvelo.

Specifically, we evaluated how well the embedding preserves cell-type information using a kNN classification accuracy with fivefold cross-validation. Given an embedding matrix in 2D or 3D space ($X\in \mathbb{R} ^{n\ast d}$, where n is the number of cells and d is 2 or 3) and corresponding cell-type labels ($y\in \left \{1,\ldots ,C\right \}$), we partition the data into fivefolds. For each fold (k), a kNN classifier with K=5, denoted as $f^{\left (k\right)}$, is trained on the training subset and evaluated on the held-out test subset. The classification accuracy for the k th fold is defined as

(15) $$\displaystyle  Acc^{\left (k\right)}=\frac{1}{n_{k}}\sum _{i\in D_{k}^{test}}1\left (f^{\left (k\right)}\left (x_{i}\right)=y_{i}\right)$$

where $n_{k}$ is the number of samples in the test set and 1(·) is the indicator function. The final score is obtained by averaging across all folds:

(16) $$\displaystyle  Acc=\frac{1}{5}\sum _{k=1}^{5}Acc^{\left (k\right)}$$

This metric directly assesses whether cells of the same type are positioned close to each other in the embedding space and is widely used to quantify representation quality.

### GO term enrichment analysis

GO terms enrichment analysis is a widely used method for identifying biological processes, molecular functions, or cellular components that are overrepresented in a given set of genes compared to a background set. Here we use the biological processes enrichment analysis in the GO terms database to explore the associated biological roles of those selected velocity genes. To perform the GO term enrichment analysis, we utilized the ‘gseapy.enrichr()’ function in Python using Fisher’s exact test by default.

### Merging of lineage-specific results

For datasets containing multiple lineages, TSvelo applies its model independently to each lineage. The resulting lineage-specific outputs are integrated into a unified representation by aggregating both expression layers and cell-level annotations across branches.

All objects are aligned to a common gene space by restricting to the shared set of genes. Let b=1,…,B index the lineage-specific results. For each cell c and each inferred quantity (e.g., velocity and pseudotime), TSvelo collects the corresponding results $x_{c,b}$ from all lineages in which the cell is present. Layer values are merged on a per-cell basis using a weighted average, where the weight for each branch is given by its number of cells, $n_{b}$. The merged value for cell c is computed as

(17) $$\displaystyle  \overset{\sim }{x}_{c}=\frac{\sum _{b}n_{b}\cdot x_{c,b}}{\sum _{b}n_{b}}$$

Continuous variables are averaged, while discrete variables are cast to integer values after aggregation.

Finally, the aggregated layer values and annotations are assigned back according to the global cell index, producing a unified dataset that integrates lineage-specific inferences.

### Code availability statement

TSvelo is implemented in Python. The source code can be downloaded from the GitHub repository, https://github.com/lijc0804/TSvelo (copy archived at Li, 2026).

## Data Availability

The pancreatic endocrinogenesis dataset comprises the single-cell RNA-seq (10X) data of pancreatic epithelial and Ngn3-Venus fusion cells sampled from mouse embryonic day 15.5, which could be loaded using scVelo's package scvelo.datasets.pancreas(). The gastrulation erythroid dataset, which is selected from the transcriptional profiles of mouse embryos39, which could be loaded using scVelo's package scvelo.datasets.pancreas(). 10x embryonic mouse brain dataset is provided at the 10x website at https://www.10xgenomics.com/resources/datasets/fresh-embryonic-e-18-mouse-brain-5-k-1-standard-1-0-0. The data preprocessed by Multivelo is utilized in this study, (https://multivelo.readthedocs.io/en/latest/MultiVelo_Fig2.html). The dentate gyrus neurogenesis data is available at http://pklab.med.harvard.edu/velocyto/DentateGyrus/DentateGyrus.loom. The LARRY dataset has been shared by pyrovelocity, which could be accessed at https://figshare.com/articles/dataset/larry_invitro_adata_sub_raw_h5ad/20780344. The raw data of Hindbrain (pons) of adolescent mice is from https://pklab.med.harvard.edu/ruslan/velocity/oligos/. The ENCODE TF-target database website: https://maayanlab.cloud/Harmonizome/dataset/ENCODE+Transcription+Factor+Targets. The ChEA TF–target database website: https://maayanlab.cloud/Harmonizome/dataset/CHEA+Transcription+Factor+Targets. The results of BayVel on the pancreas dataset are downloaded from its GitHub page at https://github.com/elenasabbioni/BayVel_notebooks/tree/main/real%20data/Pancreas/moments/output (Sabbioni, 2025). The following previously published datasets were used: QinQ 2022larry_invitro_adata_sub_raw.h5adfigshare10.6084/m9.figshare.20780344 TritschlerS 2019Comprehensive single cell mRNA profiling reveals a detailed roadmap for pancreatic endocrinogenesisNCBI Gene Expression OmnibusGSE13218810.1242/dev.17384931160421 Pijuan-SalaB GriffithsJ 2018Timecourse single-cell RNAseq of whole mouse embryos harvested between days 6.5 and 8.5 of developmentArrayExpressE-MTAB-6967 10x Genomics 2020Fresh Embryonic E18 Mouse Brain (5k)10x Genomicsfresh-embryonic-e-18-mouse-brain-5-k-1-standard-1-0-0 LinnarssonS 2016RNA-seq analysis of single cells of the oligodendrocyte lineage from nine distinct regions of the anterior-posterior and dorsal-ventral axis of the mouse juvenile central nervous systemNCBI Gene Expression OmnibusGSE75330 WeinrebC Rodriguez-FraticelliA CamargoF KleinAM 2019Lineage tracing on transcriptional landscapes links state to fate during differentiationNCBI Gene Expression OmnibusGSE14080210.1126/science.aaw3381PMC760807431974159 HochgernerH ZeiselA LönnerbergP LinnarssonS 2017Transcriptome analysis of single cells from the mouse dentate gyrusNCBI Gene Expression OmnibusGSE95753

## Additional files

MDAR checklist

## Appendix 1

**Appendix 1—table 1.** The comparison of RNA velocity approaches.

Methods	Biological scope	Key assumptions	Inference framework	Modeling granularity
velocyto	Transcription and splicing	Steady state.	Least squares regression	Per-gene
scVelo	Transcription and splicing	Two-step transcription rates. Constant splicing and degradation rates.	Expectation–maximization (EM)	Per-gene
VeloAE	Transcription and splicing	Steady state.	Auto-encoder (AE)	Joint (all genes simultaneously)
UniTVelo	Transcription and splicing	Spliced RNA abundance is a twice-differentiable function of time.	Radial basis function (RBF)	Per-gene
Dynamo	Transcription and splicing or metabolic	Two-step transcription rates. Constant splicing and degradation rates.	Ordinary differential equations (ODEs) and sparseVFC	Per-gene
cellDancer	Transcription and splicing	Cell-specific time-dependent transcription, splicing, and degradation rates.	Convolutional neural network (CNN)	Per-gene
MultiVelo	Transcription and splicing	Two-step chromatin accessibility states. Constant splicing and degradation rates.	EM	Per-gene
BayVel	Transcription and splicing	A product of Poisson distributions for the initial state.	Markov chain Monte Carlo	Per-gene
TFvelo	Regulation	Cell-specific time-dependent transcription rate. Constant degradation rates.	Generalized expectation–maximization	Per-gene
TSvelo	Regulation, transcription, and splicing	Cell-specific time-dependent transcription rates. Constant splicing and degradation rates.	Neural ODE	Joint (all genes simultaneously)

### Simulation data analysis

We generated synthetic single-cell RNA velocity datasets using a mechanistic transcriptional dynamics model with one or multiple developmental branches. The system included 200 genes, among which 30 were designated as transcription factors (TFs).

For each branch, we independently sampled a TF–target regulatory matrix $W\in \mathbb{R} ^{30\times 200}$ from a standard normal distribution to simulate distinct GRN structures. Gene expression dynamics were modeled using a coupled ordinary differential equation (ODE) system describing unspliced and spliced RNA abundances:

$$\displaystyle \frac{du_{g}\left (t\right)}{dt}=\alpha _{g}\left (t\right)- \beta _{g}u_{g}\left (t\right)$$

$$\displaystyle \frac{ds_{g}\left (t\right)}{dt}=\beta _{g}u_{g}\left (t\right)- \gamma _{g}s_{g}\left (t\right)$$

where u and s denote unspliced and spliced RNA levels, respectively. The transcription rate α was computed as a nonlinear function of TF expression, defined as a weighted sum of spliced TF abundance, followed by clipping to ensure bounded activation.

Each branch is initialized from the same randomly sampled initial condition drawn from a gamma distribution, allowing controlled divergence of trajectories driven solely by branch-specific regulatory programs.

To simulate observed sequencing counts, we introduced technical noise by scaling latent expression levels with cell-specific library sizes drawn from a log-normal distribution. The resulting expression counts were generated using a negative binomial sampling model:

$$\displaystyle X\sim NB\left (\mu =library \,size\times x,\theta \right)$$

where θ controls overdispersion, with smaller values corresponding to higher noise levels. The final datasets consist of paired unspliced (U) and spliced (S) count matrices with realistic transcriptional stochasticity and branching gene regulatory dynamics. For each branch, cells were further divided into three developmental stages for downstream analysis.

We evaluated TSvelo on multiple simulated datasets with varying numbers of branches and noise levels. There are two or three branches starting from the same root cell groups in these datasets (Branch 1: stage 0–stage 1–stage 2. Branch 2: stage 0–stage 3–stage 4. Branch 3: stage 0–stage 5–stage 6). The results of initial state identification based on the unspliced-to-spliced (U-to-S) delay, along with the corresponding 2D velocity stream visualizations, are presented in Figure 1—figure supplement 1. These results demonstrate that the U-to-S delay-based initialization is robust and consistently identifies cells corresponding to the earliest developmental stage (stage 0) across different simulation settings.

We also evaluated TSvelo and those splicing-based RNA velocity approaches on multiple simulated datasets with varying numbers of branches and noise levels. There are one, two, or three branches starting from the same cell group in these datasets (Branch 1: stage 0–stage 1–stage 2. Branch 2: stage 0–stage 3–stage 4. Branch 3: stage 0–stage 5–stage 6). We primarily assessed performance using the cross-boundary direction correctness (CBDir) metric, as it directly evaluates inferred trajectories against ground-truth cell-stage annotations. As shown in Figure 1—figure supplement 2, TSvelo consistently achieves the highest accuracy across all simulation settings, particularly in scenarios with complex branching structures, which pose significant challenges for baseline methods.

### The U-to-S delay scores for each Leiden cluster when treated as the initial state across all datasets

Along a correctly oriented trajectory, unspliced (U) expression is expected to precede spliced (S) expression due to transcriptional dynamics. Ideally, this U-to-S delay would be observable at the level of individual genes. However, due to the high noise inherent in scRNA-seq data, such delays are often not consistently detectable on a per-gene basis. To address this, we aggregate U-to-S delay signals across all genes and determine the lineage orientation by maximizing a global delay score. Under this criterion, the cluster from which all outgoing lineages exhibit the highest aggregated U-to-S delay is inferred to correspond to the initial state. The results on all datasets (Figure 1—figure supplement 3) suggest that the highest U-to-S delay scores can be used to detect the initial cluster.

### Ablation studies on the selection of TF–target resources

we additionally incorporated the DoRothEA regulon database as an alternative prior with confidence-level filtering. We further performed ablation studies on the pancreas dataset and the gastrulation erythroid dataset using different TF–target resources, including ChEA, ENCODE, and their combinations with DoRothEA.

The results on the pancreas dataset and the gastrulation erythroid dataset are shown in Figure 1—figure supplements 4 and 5, respectively, which come up with the same conclusion. We observed highly consistent results across most TF–target prior combinations, including ChEA, ENCODE, ChEA + ENCODE, ChEA + DoRothEA, ENCODE + DoRothEA, and ChEA + ENCODE + DoRothEA. Using the pancreas dataset as an example, the mean velocity consistency ranged from 0.985 to 0.995, the mean in-cluster coherence ranged from 0.983 to 0.992, and the mean cross-boundary direction correctness ranged from 0.719 to 0.740 across all settings. These consistently high and tightly bounded metrics indicate that TSvelo is largely insensitive to the specific choice of TF–target prior.

The only configuration showing reduced stability was the use of DoRothEA alone, particularly in terms of cross-boundary direction correctness. This is likely due to its comparatively limited coverage of TF–target interactions. For instance, in the pancreas dataset, only 81 out of 2000 highly variable genes (HVGs) could be associated with TFs based on DoRothEA, corresponding to 102 TF–target links in total, which may restrict downstream regulatory modeling. In contrast, ChEA covered 1793 genes with 13,976 TF–target links, and ENCODE covered 1854 genes with 33,076 links.

These results further suggest that integrating multiple TF–target resources can improve performance by increasing regulatory coverage and reducing false negatives in the prior network. Given that unsupported edges can be down-weighted during training, TSvelo appears to be more sensitive to missing true regulatory interactions than to the inclusion of spurious ones.

### Computational benchmark

we have added a systematic comparison of runtime and GPU memory usage across TSvelo and ba methods using simulated datasets of increasing scale (600, 1200, and 1800 cells) on our NVIDIA GeForce RTX 3090 device with 24 GB memory.

Appendix 1—table 2 shows differences in computational efficiency and resource requirements among methods. Specifically, classical methods such as scVelo and Dynamo exhibit very fast runtimes (10–24 s) and do not rely on GPU acceleration, reflecting their relatively lightweight modeling strategies. In contrast, deep learning-based approaches, including UniTVelo, cellDancer, and TSvelo, have higher computational costs due to their increased model complexity.

TSvelo exhibits a stable GPU memory footprint (~1.26 GB) across different dataset sizes, indicating that its memory usage is primarily determined by model architecture rather than the number of cells. This level of memory consumption is well within the capacity of modern GPUs and does not pose practical limitations. In terms of runtime, TSvelo scales approximately linearly with dataset size. The higher computational cost of TSvelo is mainly due to its EM-style optimization procedure, where each M-step also involves multiple optimization updates to infer gene regulatory effects in a global model. This design enables TSvelo to explicitly incorporate regulatory priors and jointly model gene interactions, which is not supported by these baseline methods.

**Appendix 1—table 2.** Comparison of memory and runtime cost of different methods.

	Dataset scale	600 cells	1200 cells	1800 cells
scVelo	GPU memory	-	-	-
	Modeling time	10 s	12 s	14 s
Dynamo	GPU memory	-	-	-
	Modeling time	24 s	20 s	24 s
cellDancer	GPU memory	-	-	-
	Modeling time	82 s	118 s	245 s
UniTVelo	GPU memory	930 MB	994 MB	1122 MB
	Modeling time	345 s	637 s	1016 s
TSvelo	GPU memory	1262 MB	1264 MB	1264 MB
	Modeling time	734 s	1544 s	3027 s

To further improve runtime efficiency, TSvelo allows flexible control of the number of EM iterations. As shown in Figure 1—figure supplement 6 and Appendix 1—table 3, we evaluated performance under different iteration settings on the simulation dataset. The early stopping strategy employed in the EM framework of TSvelo, which will stop modeling if the loss is not further reduced in the last three iterations. Results show that convergence is typically achieved within three iterations for this dataset, and increasing the maximum number of iterations beyond this does not further change the results. Notably, even a single iteration already yields competitive performance, likely benefiting from the strong initialization based on unspliced-to-spliced temporal delay.

Overall, these results highlight a trade-off between computational efficiency and modeling expressiveness. While TSvelo is more computationally demanding than classical approaches, it provides a more flexible framework for incorporating regulatory information and capturing complex gene interactions, which we believe justifies the additional computational cost in scenarios requiring accurate dynamical inference.

**Appendix 1—table 3.** Runtime of TSvelo under different numbers of expectation–maximization (EM) iterations on the simulation dataset. The results demonstrate that TSvelo achieves stable performance even with a small number of iterations. Due to the early stopping mechanism of the EM framework, since TSvelo gets the best results at iteration 3, it actually performs no more than six iterations.

Number of max iterations set in hyperparameters	1	2	3	4	5
Running time (s)	1007	1168	2145	2208	2989
Number of max iterations set in hyperparameters	6	7	8	9	10
Running time (s)	3120	3256	3126	3121	3027

### Null simulations for evaluating RNA velocity approaches on data without underlying dynamic structure

We have added null simulations to evaluate TSvelo and baseline approaches on data without underlying dynamic structure. Specifically, we generated a null dataset including 200 genes and 600 cells by independently sampling spliced (S) and unspliced (U) counts, thereby removing any coherent transcriptional relationship between them.

When applying scVelo and UniTVelo to this data, no genes passed the velocity gene selection step under the default likelihood-based filtering, and no velocity field could be obtained. We further tested TSvelo, Dynamo, and cellDancer on the same null data and observed that all three methods still produce trajectory-like patterns despite the absence of true dynamics (Figure 1—figure supplement 7).

Including TSvelo, many RNA velocity and trajectory inference approaches assume that they are applied to datasets reflecting underlying dynamic biological processes. Incorporating additional checks during preprocessing could help prevent applying velocity analysis to non-dynamic datasets.

### Additional results on pancreas dataset

We conducted comparisons across all methods on the pancreas dataset, with quantitative evaluations shown in Figure 2—figure supplement 1. In each plot, methods are ranked in descending order of their mean values. Numbers at the bottom indicate the sample size for each metric. Statistical significance is assessed using a one-sided Mann–Whitney *U* test, where *****, ***, **, and * denote p < 0.00001, 0.0001 ≤ p < 0.001, 0.001 ≤ p < 0.01, and 0.01 ≤ p < 0.05, respectively.

TSvelo significantly outperforms all baseline methods in terms of velocity consistency. For in-cluster coherence, TSvelo performs comparably to the best-performing baselines (UniTVelo and TFvelo) and significantly outperforms several competing methods, including CellDancer, Dynamo, and scVelo. For cross-boundary direction correctness, TSvelo shows consistent improvements in mean performance. The pairwise comparisons on cross-boundary direction correctness do not reach statistical significance. It is likely influenced by the limited number of independent samples (*n* = 7), which reduces statistical power for detecting differences. Importantly, TSvelo still achieves the best average performance among all methods, indicating a consistent overall trend in favor of TSvelo.

TSvelo is capable of accurately modeling the dynamics of spliced mRNA abundance for those TFs which are not selected as velocity genes during preprocessing. These TFs cannot be well modeled in the unspliced–spliced phase portrait. However, TSvelo overcomes this limitation by directly modeling the process from transcription to spliced mRNA. Figure 2—figure supplement 3 shows the modeling on those TFs on pancreas dataset, where TSvelo directly models the dynamic between transcription and spliced abundance.

### Additional results on gastrulation erythroid dataset

In the conventional 2D u–s phase portrait, cells from different transcriptional states may overlap, leading to reduced separability. In contrast, introducing the latent variable α expands the representation to a 3D space, which helps disentangle these mixed states and yields a clearer phase structure. We provide quantitative evidence on this gastrulation erythroid dataset in Figure 3—figure supplement 1, showing that the 3D representation achieves consistently higher kNN classification accuracy for cell-state separation compared to the 2D u–s embedding (one-sided Mann–Whitney *U* test, p-value = 0.002).

Figure 3—figure supplement 2 shows the dynamics fitting on additional genes with TSvelo on the gastrulation erythroid dataset.

### Additional results on mouse brain dataset

Figure 4—figure supplement 1 shows the dynamics fitting on additional genes with TSvelo on the mouse brain dataset.

### Comparison between the chrome accessibility rate used in MultiVelo and the learned transcription rate from TSvelo

We have conducted the requested analysis by computing gene-wise chrome accessibility rate used in MultiVelo and the learned transcription rate from TSvelo, and evaluated their correlation across genes. As shown in Figure 4—figure supplement 2, the two estimates exhibit almost no global correlation across genes, indicating that they capture substantially different aspects of regulatory information.

This discrepancy is not unexpected and reflects the fundamental differences between these modalities. scATAC-seq measures chromatin accessibility, which provides a proxy for cis-regulatory potential of genomic regions. However, ATAC signals are inherently sparse and often exhibit a near-binary structure, limiting their ability to directly capture fine-grained temporal regulatory dynamics. In contrast, TF RNA expression reflects downstream transcriptional output, which is shaped by multiple regulatory layers, including post-transcriptional regulation, protein activity, temporal delays, and indirect regulation through intermediate transcriptional or signaling pathways. As a result, these two modalities are expected to capture complementary but not directly comparable aspects of gene regulation.

Overall, this result suggests that ATAC- and TF RNA-based signals capture distinct aspects of gene regulation. This further implies that integrating both modalities may be beneficial for future models that aim to more comprehensively characterize transcriptional regulation.

### TSvelo can accurately model gene dynamics and cell fate on the pons dataset

The pons dataset originates from the hindbrain of adolescent mice and captures the differentiation pathway from oligodendrocyte precursor cells (OPCs) to committed oligodendrocyte precursor cells (COPs), progressing to newly formed oligodendrocytes, and finally culminating in myelin-forming oligodendrocytes. The velocity genes selected through TSvelo’s preprocessing strategies are mostly enriched in Gene Ontology (GO) terms related to the development of the nervous system, including regulation of axon extension involved in axon guidance (GO:0048841), axonogenesis (GO:0007409), and nervous system development (GO:0007399) (Figure 4—figure supplement 3a).

Using the unspliced-to-spliced delay, TSvelo can correctly detect the initial Leiden cluster on the pons dataset (Figure 4—figure supplement 3b, c). After performing the model optimization for the neural ODE system, the pseudotime and velocity learned by TSvelo can accurately fit the differentiation process in the data (Figure 4—figure supplement 3d, e). Figure 4—figure supplement 3f illustrates the dynamics of multiple genes modeled by TSvelo. A challenge in this dataset is the abrupt shift in gene expression between OPCs (red) and COPs (orange). For example, as shown in Figure 4—figure supplement 3f for CHN2 and DPYSL2, there is a significant gap in expression levels between OPCs and COPs. Despite this, TSvelo successfully captures their dynamic patterns.

### The lineages segmentation and combination on the dentate gyrus dataset

Using the dentate gyrus dataset, we demonstrate how lineages are segmented during preprocessing, and subsequently combined during postprocessing after being modeled individually. Notably, the lineage segmentation process is fully automated and does not require prior knowledge about the presence of multiple branches.

First, Leiden clustering is performed on the preprocessed scRNA-seq data, with the resolution set to 0.1 by default (Figure 5—figure supplement 1a). A cluster–cluster graph is then constructed using PAGA (Figure 5—figure supplement 1b), followed by a filtering strategy that removes edges with weights below a threshold of 0.02 by default. Next, each Leiden cluster is treated as an initial cluster, and the appropriate initialization is determined using the unspliced to spliced delay.

For instance, using Leiden cluster 3 (colored in red at Figure 5—figure supplement 1a) as the initial cluster, diffusion pseudotime (DPT) is initialized with the selected cluster (Figure 5—figure supplement 1c). Starting from this initial cluster, the shortest paths to all other clusters are computed. Any path that is a subset of another is discarded. The remaining paths correspond to the detected lineages. In the dentate gyrus dataset, initializing with Leiden cluster 3 yields three distinct lineages (Figure 5—figure supplement 1d). By applying DPT to each lineage, we can calculate the U-to-S delay for each lineage and determine the overall U-to-S delay for this initialization with the selected cluster.

After calculating the U-to-S delay for each Leiden cluster initialization, the best initialization cluster is chosen based on the highest U-to-S delay (Figure 5—figure supplement 1e). The corresponding DPT is then used to initialize pseudotime in the downstream neural ODE model. Subsequently, each lineage is processed independently in the TSvelo for optimization, yielding both the optimized pseudotime and velocity model. The results for each lineage are shown in Figure 5—figure supplement 1f.

Finally, on the dataset where multiple branches are detected, to combine the models for all lineages and obtain the overall pseudotime and velocity stream, we compute the average velocity (Figure 5—figure supplement 1g) and pseudotime (Figure 5—figure supplement 1h) across the different lineages. For example, in the dentate gyrus dataset, since Leiden clusters 0, 1, 2, and 4 each belong to a single lineage, the velocity and pseudotime values calculated for these lineages are directly assigned to the combined file. For Leiden cluster 3, which is included in all three branches, the mean velocity and mean pseudotime across the three lineages are calculated for cells in this cluster.

### Additional results on dentate gyrus dataset

Figure 5—figure supplement 2 shows the detailed dynamics fitting on each lineage for *Ank3*, *Map1b*, and *Slc1a2* on the dentate gyrus dataset.
