# Supplementary material for: Comprehensive RNA velocity by modeling the cascade of gene regulation, transcription, and splicing from single-cell RNA sequencing data with TSvelo
Source: eLife. 2026 Sep 15;14:RP108950. doi: 10.7554/eLife.108950 (PMC13577664; doi:10.7554/eLife.108950)
Supplement: Peer review history

# Editor report: eLife Assessment

**DOI:** 10.7554/eLife.108950.4.sa0

**Authors:** Yongliang Yang

**Affiliations:** Shanghai University of Medicine and Health Sciences China

**Keywords:** Solid, Valuable

This study presents a **valuable** RNA velocity solution which integrates cell differentiation and gene regulation, with a balance between neuralODE and raw gene space. The evidence supporting the claims of the authors is **solid**, although inclusion of discussion on the challenges in capturing cell cycle transitions would have strengthened the study. The work will be of interest to scientists working in the field of computational biology and gene regulation.

# Referee report: Reviewer #1 (Public review)

**DOI:** 10.7554/eLife.108950.4.sa1

Summary:

In the paper, the authors propose a new RNA velocity method, TSvelo, which predicts the transcription rate linearly based on the expression of RNA levels of transcription factors. This framework is an extension of its recent work TFvelo by including unspliced reads and designing a coherent neuralODE framework. Improved performance was demonstrated in six diverse datasets.

Strengths:

Overall, this method introduces innovative solutions to link cell differentiation and gene regulation, with a balance between model complexity (neuralODE) and interpretability (raw gene space).

Comments on revised version:

I thank the authors for further revision, and I do not have any other concerns. I believe it is an important contribution to this field of trajectory inference and gene regulation.

# Referee report: Reviewer #3 (Public review)

**DOI:** 10.7554/eLife.108950.4.sa2

Despite the abundance of RNA velocity tools, there are still major limitations, and there is strong skepticism about the results these methods lead to. In this paper, the authors try to address some limitations of current RNA velocity approaches by proposing a unified framework to jointly infer transcriptional and splicing dynamics. The method is then benchmarked on 6 real datasets against the most popular RNA velocity tools.

Comments on revised version:

The Authors addressed my 2 follow-up comments suitably.

Thanks for the time you took addressing them. I have no further comments.

# Author comment: Author response

**DOI:** 10.7554/eLife.108950.4.sa3

**Authors:** Jiachen Li, Zhe Wang, Hong-Bin Shen, Ye Yuan

**Affiliations:** Institute of Process Engineering Beijing China; Institute of Process Engineering Beijing China; Shanghai Jiao Tong University Shanghai China; Institute of Process Engineering Beijing China

The following is the authors’ response to the previous reviews

**Public Reviews:**

**Reviewer #1 (Public review):**

Summary:

In the paper, the authors propose a new RNA velocity method, TSvelo, which predicts the transcription rate linearly based on the expression of RNA levels of transcription factors. This framework is an extension of its recent work TFvelo by including unspliced reads and designing a coherent neuralODE framework. Improved performance was demonstrated in six diverse datasets.

Strengths:

Overall, this method introduces innovative solutions to link cell differentiation and gene regulation, with a balance between model complexity (neuralODE) and interpretability (raw gene space).

Comments on revised version:

The authors have added comprehensive analyses in this revision, and all of my concerns have been very well addressed. Here, I just want to re-emphasize the original points 1 and 3.

(1) The analysis and clarification are very helpful - thanks! I found that Fig. R1 and R2 are very insightful, as DoRothEA-only returns much worse performance. Please consider adding these two figures to the supp figure and possibly highlighting your setting for edge pruning (down-weights); therefore, the model is more likely to be affected by false negatives than false positives in the TF-target prior.

We thank the reviewer for the positive feedback and for recognizing the value of the additional analyses. We have added the previous Fig. R1 and Fig. R2 to the Supplementary Information as Fig. S13 and Fig. S14, respectively, and have referred to them in the revised manuscript.

We have also expanded the description of the TF–target prior used in TSvelo in the “Acquiring Prior Knowledge of Gene Regulatory Relations” subsection of the Methods. As noted by the reviewer, TSvelo is expected to be less sensitive to false-positive TF–target interactions because unsupported edges can be down-weighted during training. In contrast, missing true regulatory interactions are not represented in the prior network and therefore cannot contribute to the learned regulatory dynamics, making the model potentially more sensitive to false negatives.

(3) Please consider adding some discussion on the challenges in capturing cell cycle transitions.

We thank the reviewer for this suggestion. We have added a brief discussion in the Discussion section on the challenges of modeling cell-cycle transitions. In particular, cell-cycle progression is often characterized by cyclic dynamics and overlapping transcriptional programs, which can complicate the inference of directional state transitions and regulatory relationships.

**Reviewer #3 (Public review):**

Despite the abundance of RNA velocity tools, there are still major limitations, and there is strong skepticism about the results these methods lead to. In this paper, the authors try to address some limitations of current RNA velocity approaches by proposing a unified framework to jointly infer transcriptional and splicing dynamics. The method is then benchmarked on 6 real datasets against the most popular RNA velocity tools.

Comments on revised version.

The Authors addressed all my comments suitably. I'd like to thank them for the time they spent addressing them: the revised paper is much more convincing.

I have 2 very minor follow-up concerns:

(1) I appreciated the simulation study, however, no null simulation is present.

We know RNA velocity tools are inclined to provide false positives: trajectories even when the data doesn't have any.

I'd be helpful to add null simulations where the data has no trajectories and see if methods erroneously identify any.

We thank the reviewer for this helpful suggestion. We have added null simulations to evaluate TSvelo and baseline approaches on data without underlying dynamic structure. Specifically, we generated a null dataset including 200 genes and 600 cells by independently sampling spliced (S) and unspliced (U) counts, thereby removing any coherent transcriptional relationship between them.

When applying scVelo and UniTVelo to this data, no genes passed the velocity gene selection step under the default likelihood-based filtering, and no velocity field could be obtained. We further tested TSvelo, Dynamo, and cellDancer on the same null data and observed that all three methods still produce trajectory-like patterns despite the absence of true dynamics (See Supplementary Information as Fig. S17).

Including TSvelo, many RNA velocity and trajectory inference approaches assume that they are applied to datasets reflecting underlying dynamic biological processes. We agree that incorporating additional checks during preprocessing could help prevent applying velocity analysis to non-dynamic datasets. We have added this discussion to the revised manuscript.

(2) Several of the novel analyses are only reported in the Supplementary material and only references in the main text (e.g., "A validation of TSvelo on simulated data is provided in Fig. S1 and Fig. S2 in the Supplementary Information."). This is pity!

If allowed, I'd add some comments about the new analyses (simulations, computational benchmarks, etc...) also in the main text.

We thank the reviewer for this suggestion. We agree that several analyses presented in the Supplementary Information provide important support for our conclusions. To improve their visibility, we have expanded the corresponding descriptions in the main text and briefly summarized the key findings of the relevant Supplementary Figures instead of only citing them. These revisions have been made for Fig. S1, Fig. S2, Fig. S10, Fig. S12, Fig. S13, Fig. S14 and Fig. S17. In particular, we have incorporated a summary of the simulation results at the end of the subsection “Estimate RNA Velocity with TSvelo” in the Results section, and added a discussion of the computational benchmarking analyses in the Discussion section. We hope these changes improve the accessibility of these results while maintaining a concise presentation of the main findings.

**Recommendations for the authors:**

**Reviewer #3 (Recommendations for the authors):**

I suggest the paper to undergo (very) minor revisions as detailed in the Public Review.

Simone Tiberi, The University of Bologna

We sincerely thank all reviewers for their thoughtful suggestions, which have helped improve the clarity and overall presentation of the manuscript.
